# Supplementary material for: Atom Economical Multi-Substituted Pyrrole Synthesis from Aziridine
Source: Molecules. 2022 Oct 13;27(20):6869. doi: 10.3390/molecules27206869 (PMC9609358; doi:10.3390/molecules27206869)

Supporting Information

Atom economical multi-substituted pyrrole synthesis from aziridine

Lingamurthy Macha, Ranjith Jala, Sang-Yun Na and Hyun-Joon Ha*

Department of Chemistry, Hankuk University of Foreign Studies, Yongin 17035, Korea

E-mail: hjha@hufs.ac.kr (H.-J. Ha)

Table of Contents:

Copies of ^{1}H and ^{13}C NMR spectra……………………………………….............p S02-S25

^{1}H NMR spectrum of (R)-2-(azidomethyl)-1-(1-phenylethyl)-5-propyl-1H-pyrrole (2a):

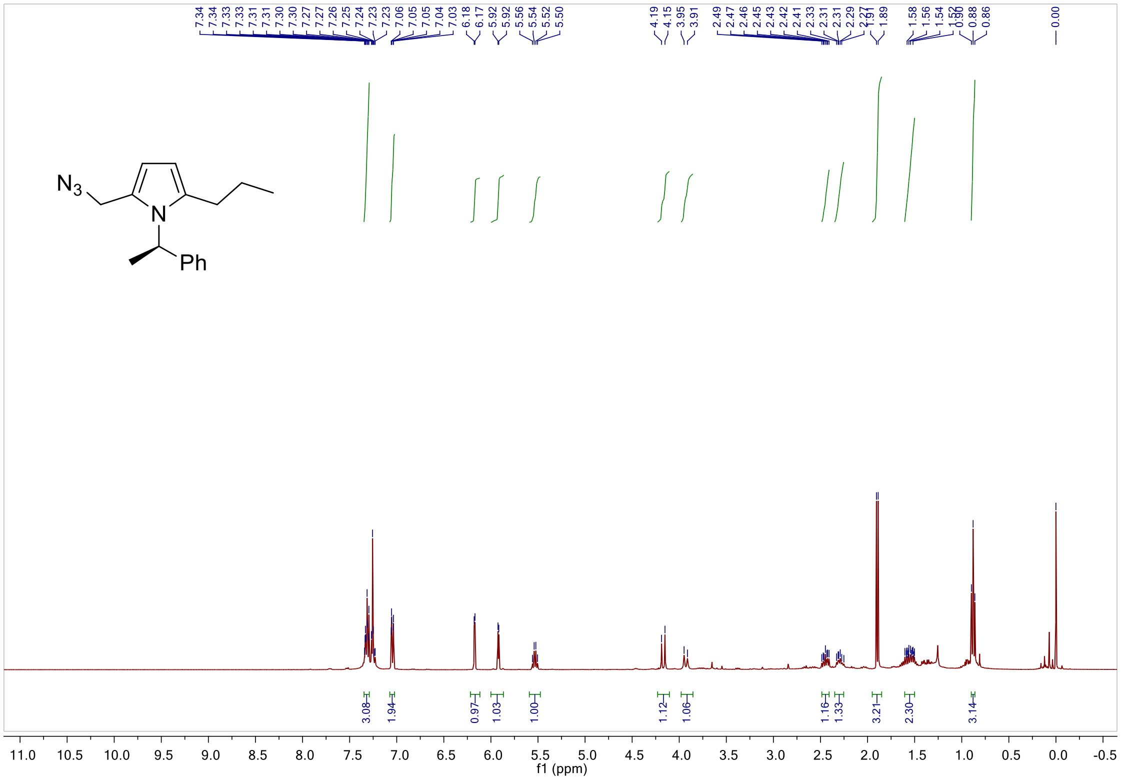

^{13}C NMR spectrum of (R)-2-(azidomethyl)-1-(1-phenylethyl)-5-propyl-1H-pyrrole (2a):

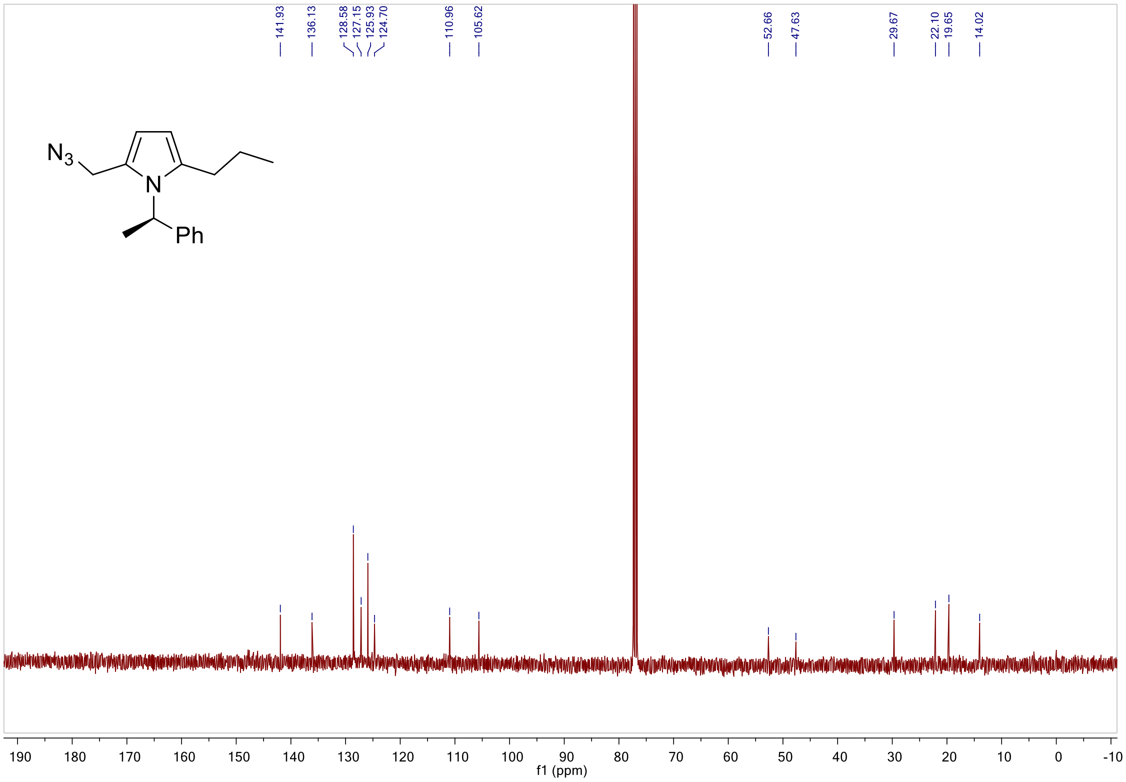

^{1}H NMR spectrum of 2-(azidomethyl)-1-benzyl-5-phenyl-1H-pyrrole (2b):

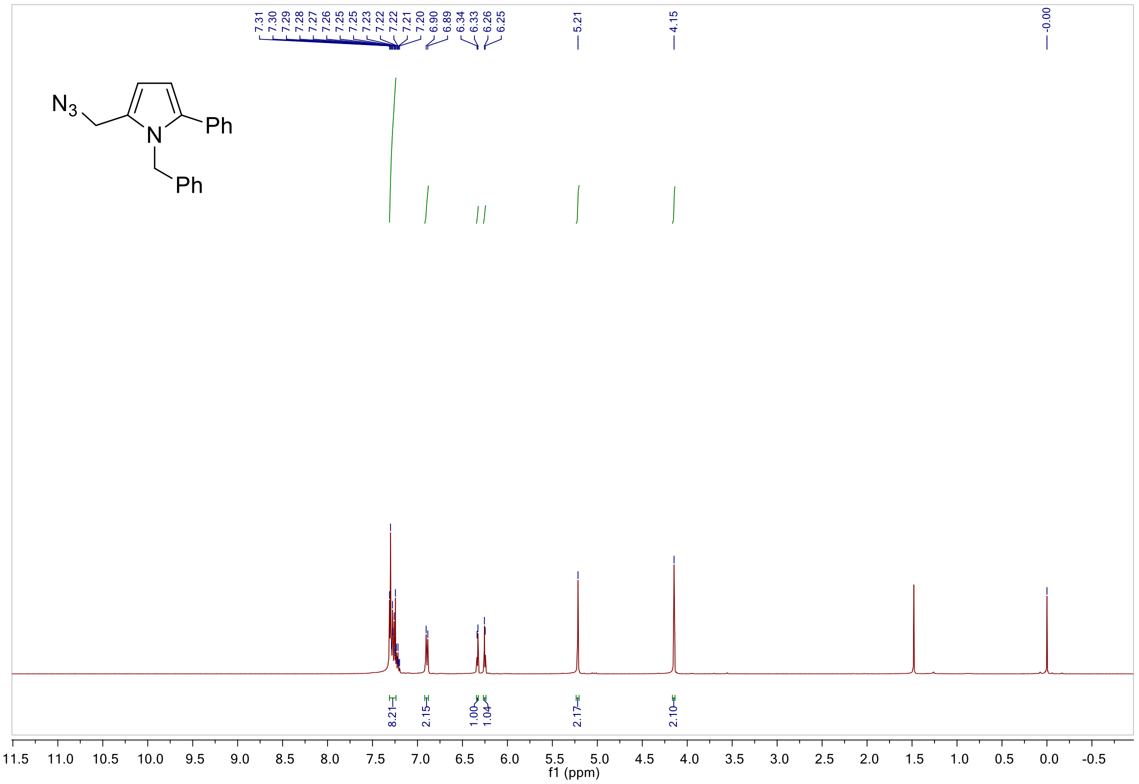

^{13}C NMR spectrum of 2-(azidomethyl)-1-benzyl-5-phenyl-1H-pyrrole (2b):

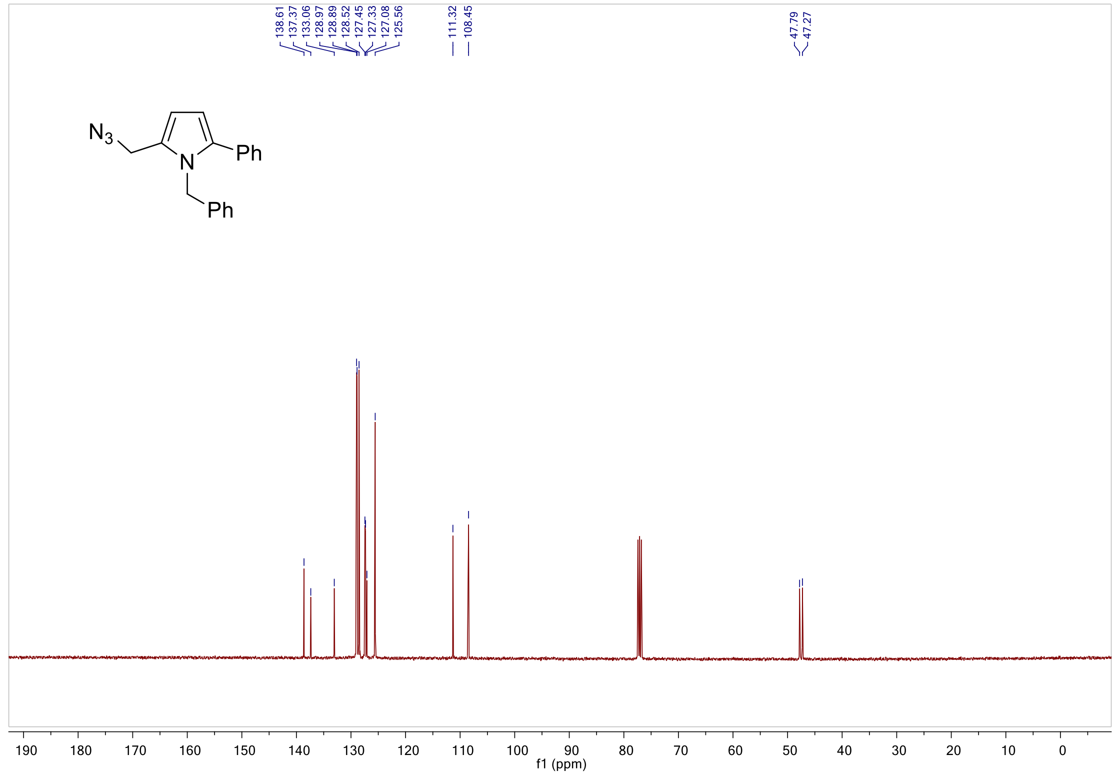

^{1}H NMR spectrum of 2-(azidomethyl)-1-benzyl-5-(2-methoxyphenyl)-1H-pyrrole (2e):

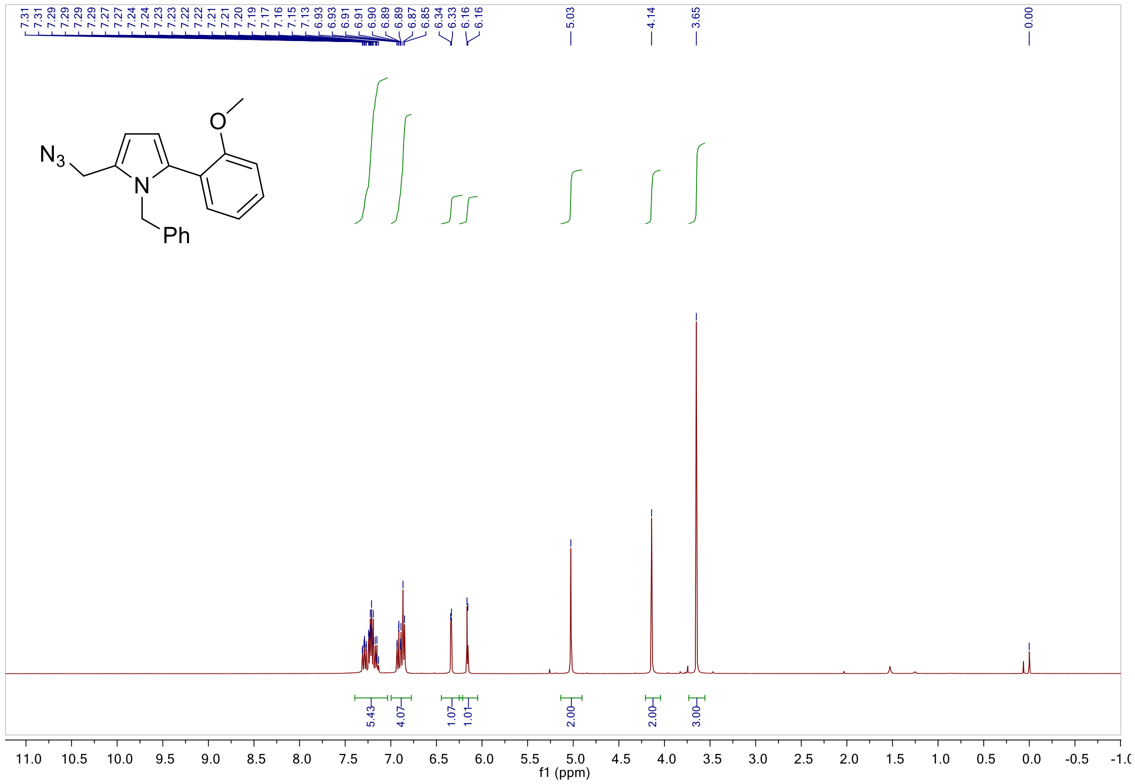

^{13}C NMR spectrum of 2-(azidomethyl)-1-benzyl-5-(2-methoxyphenyl)-1H-pyrrole (2e):

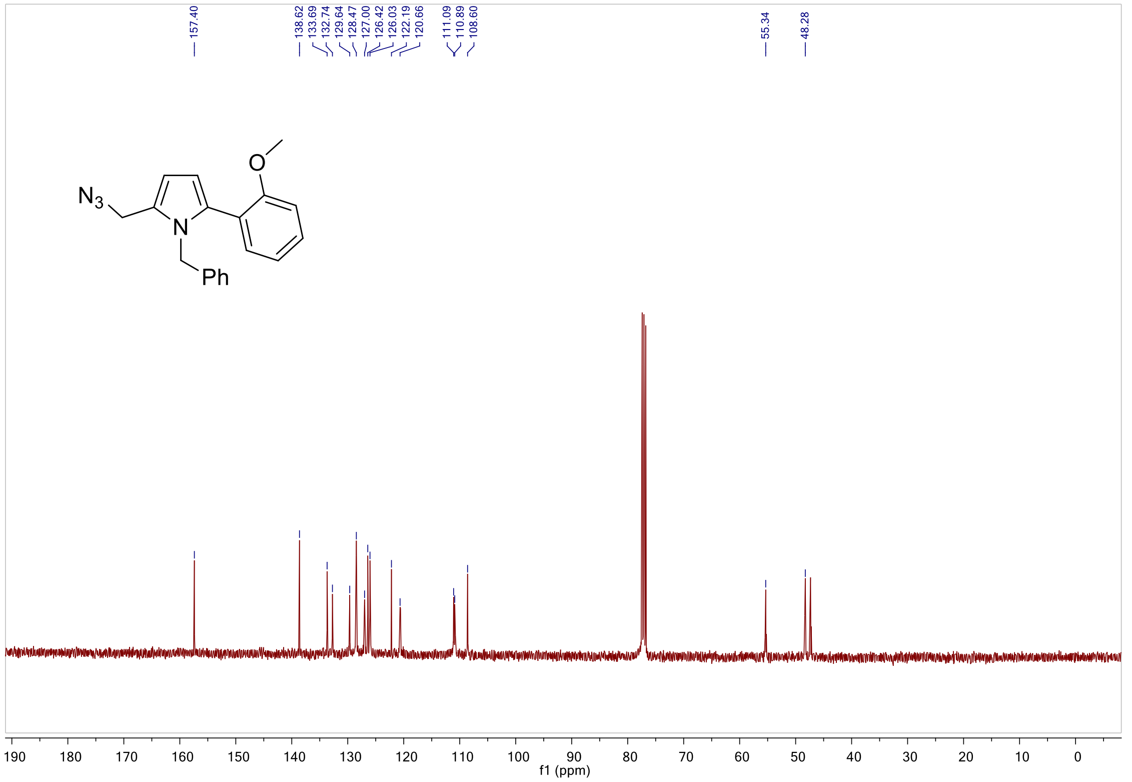

^{1}H NMR spectrum of 2-(azidomethyl)-1-benzyl-5-(4-methoxyphenyl)-1H-pyrrole (2f):

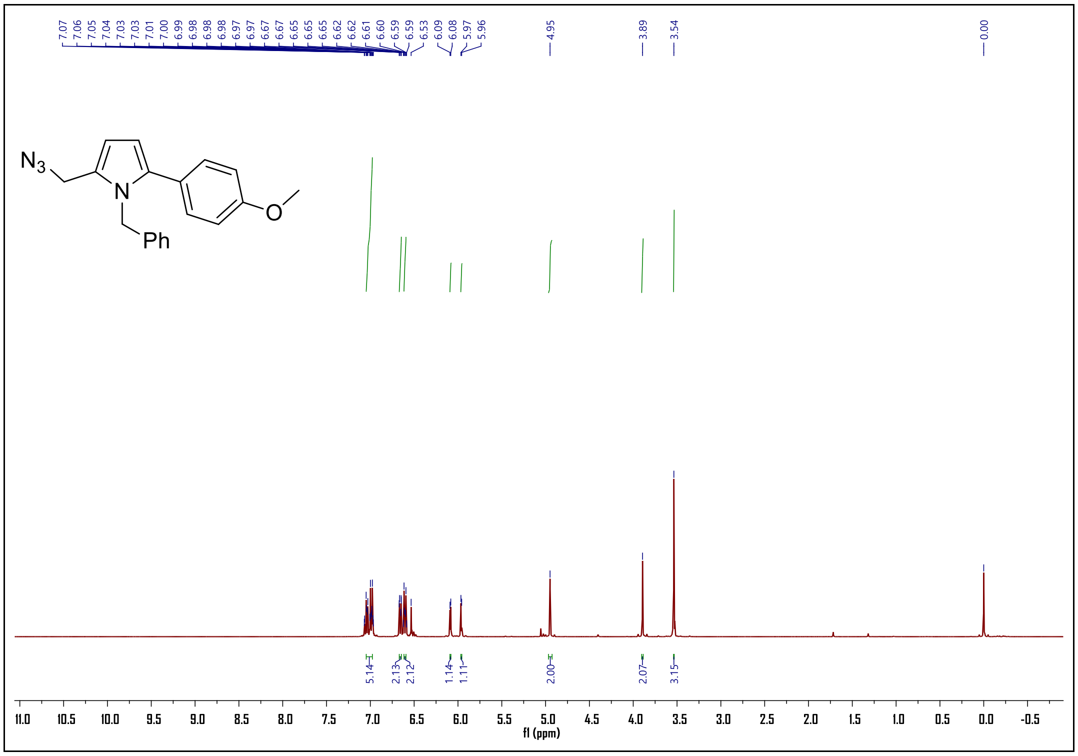

^{13}C NMR spectrum of 2-(azidomethyl)-1-benzyl-5-(4-methoxyphenyl)-1H-pyrrole (2f):

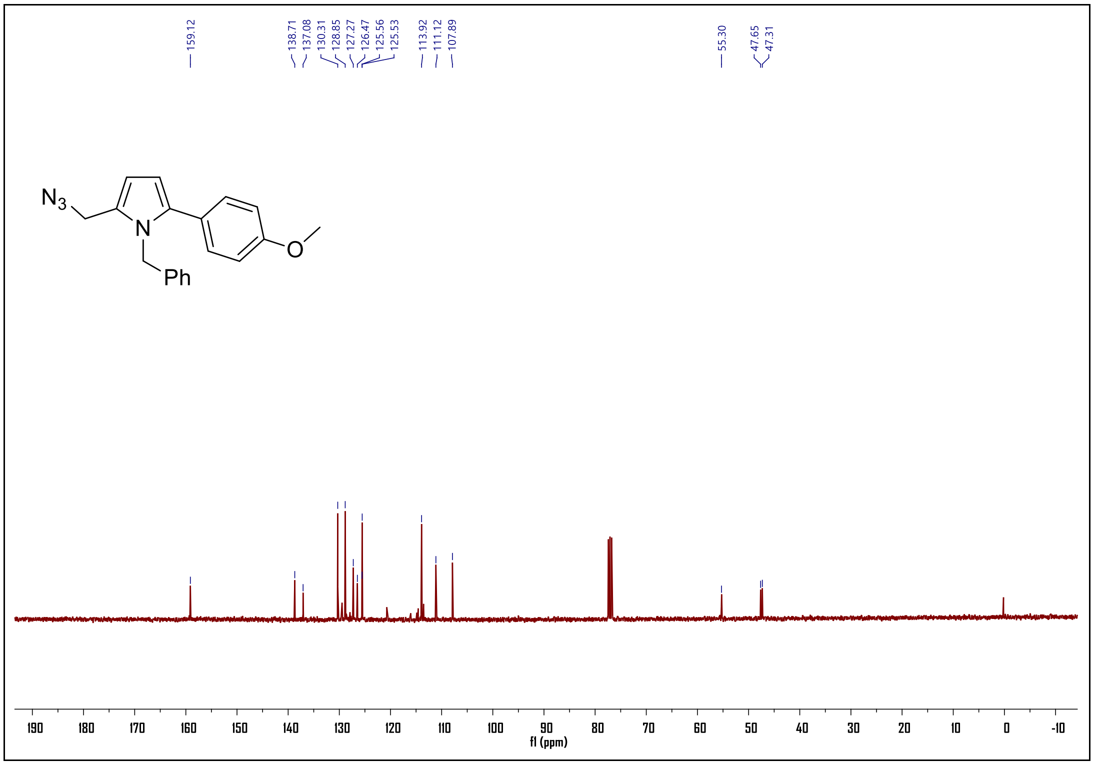

¹H NMR spectrum of 2-(azidomethyl)-1-benzyl-5-nonyl-1H-pyrrole (2g):

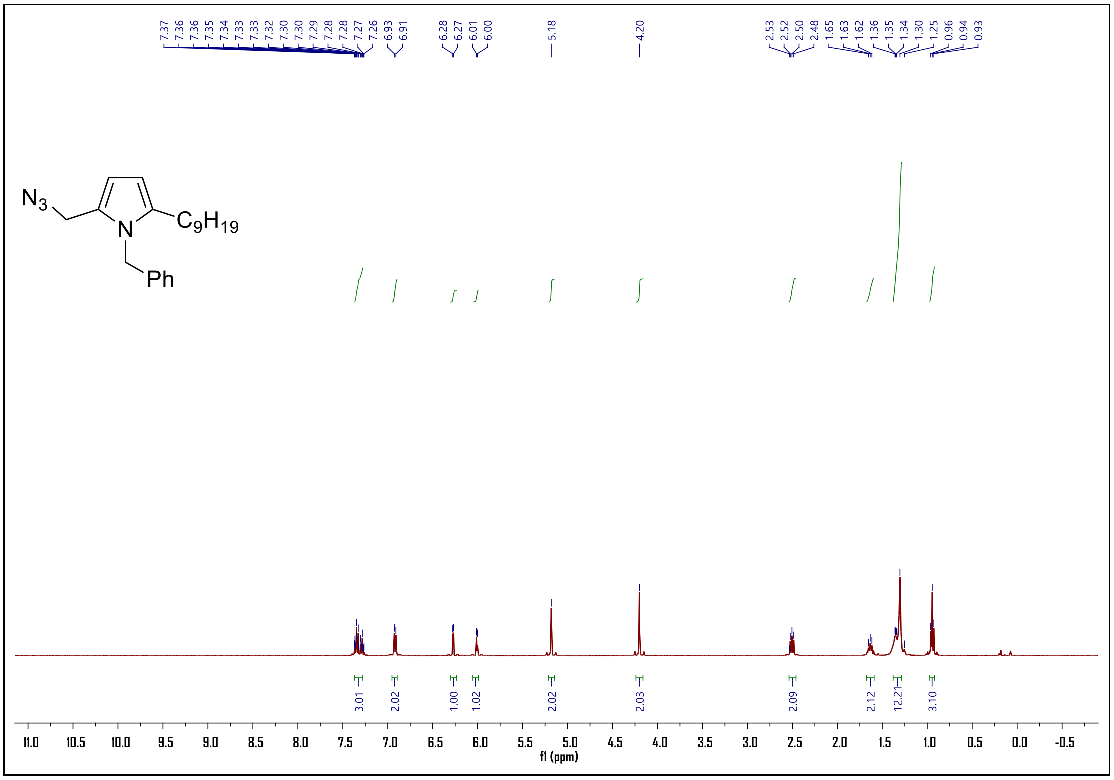

¹³C NMR spectrum of 2-(azidomethyl)-1-benzyl-5-nonyl-1H-pyrrole (2g):

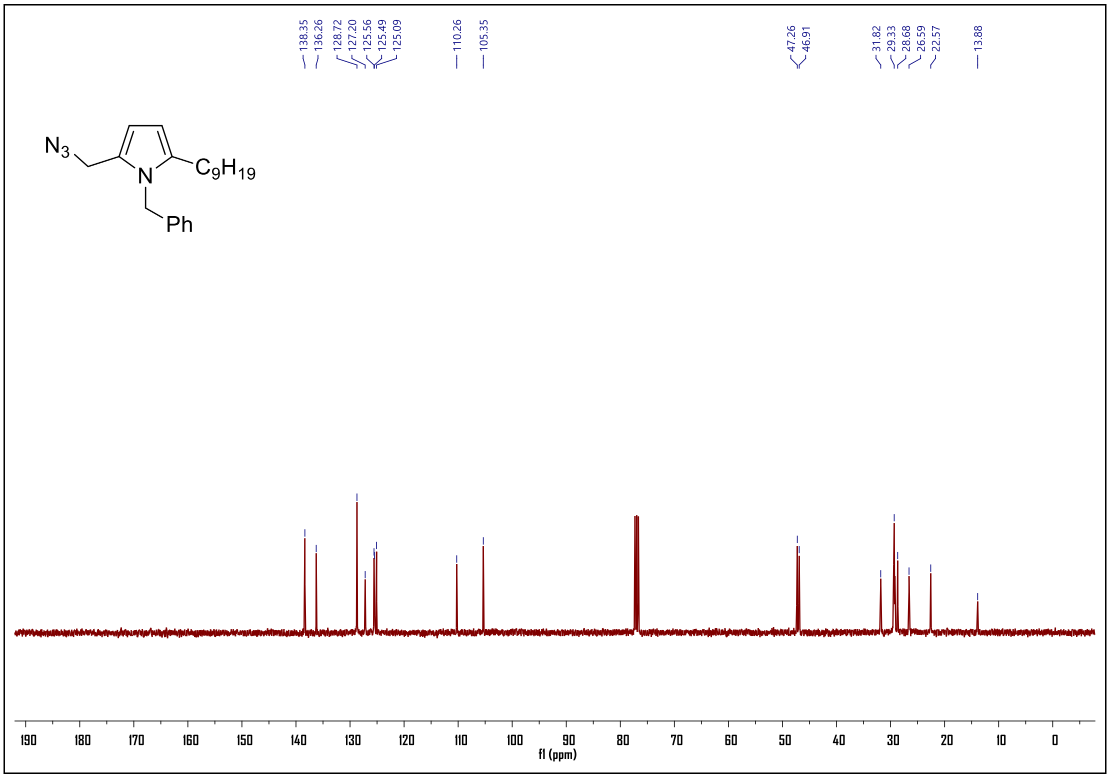

^{1}H NMR spectrum of (R)-2-(azidomethyl)-3-methyl-5-phenyl-1-(1-phenylethyl)-1H-pyrrole (2h):

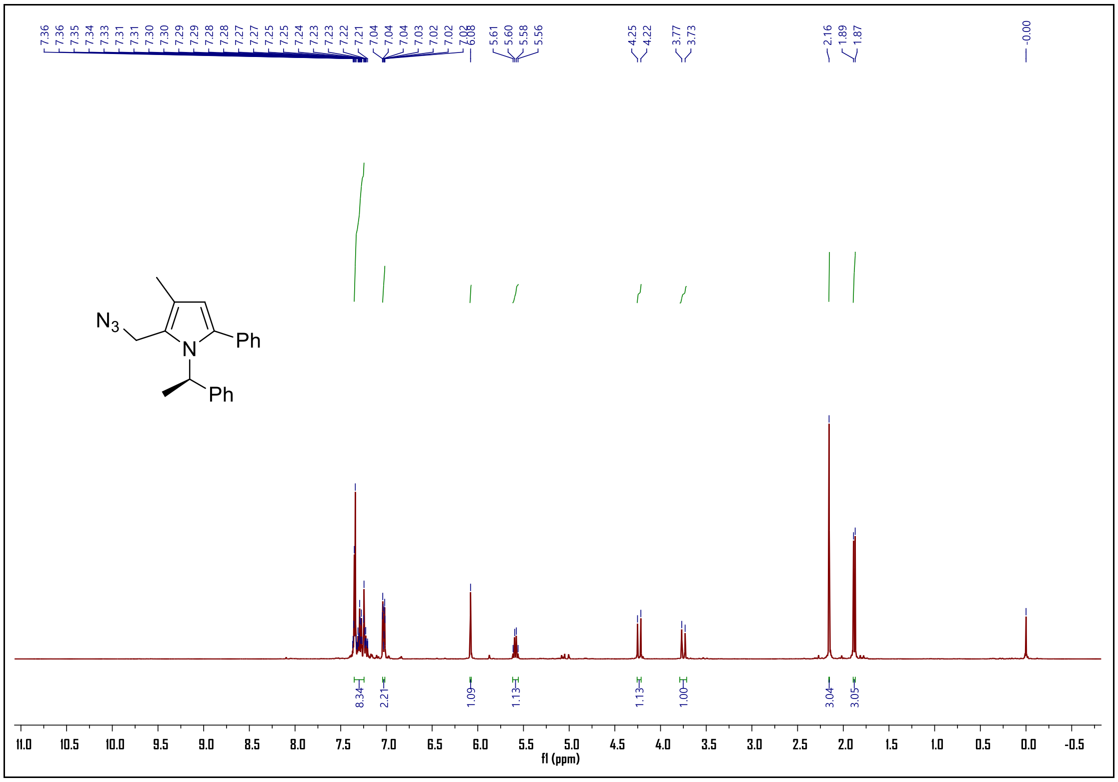

^{13}C NMR spectrum of (R)-2-(azidomethyl)-3-methyl-5-phenyl-1-(1-phenylethyl)-1H-pyrrole (2h):

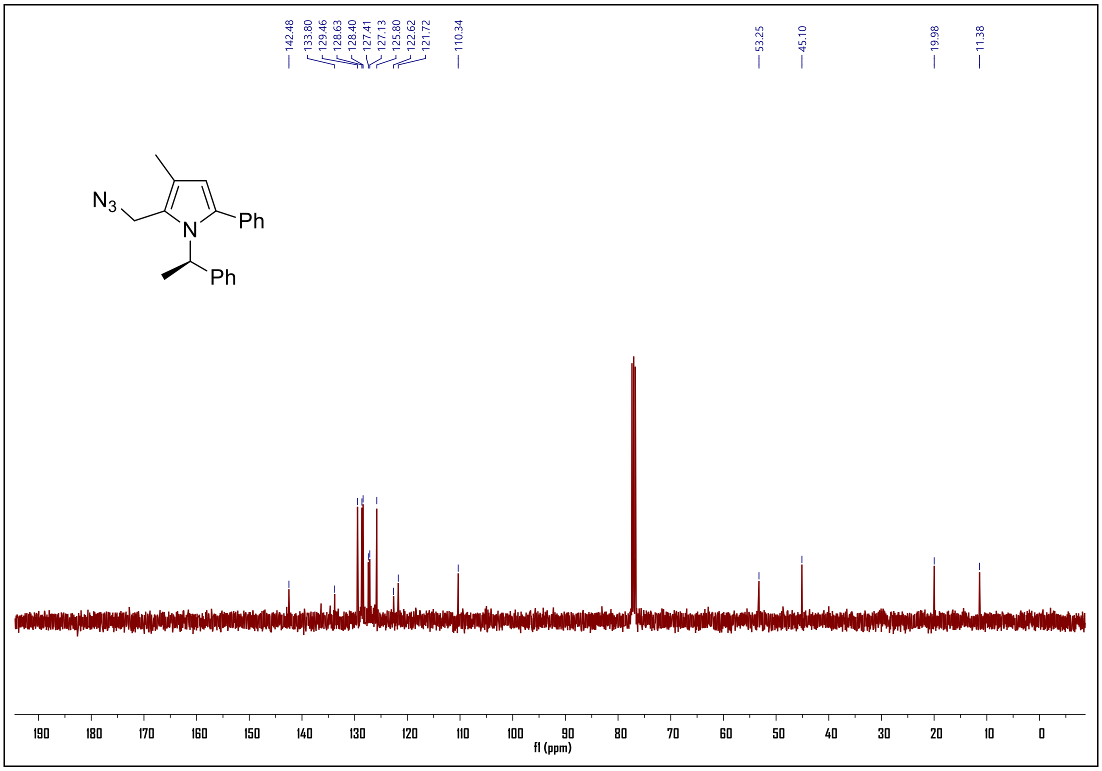

^{1}H NMR spectrum of (R)-2-(azidomethyl)-5-(((tert-butyldimethylsilyl)oxy)methyl)-3-methyl-1-(1-phenylethyl)-1H-pyrrole (2i):

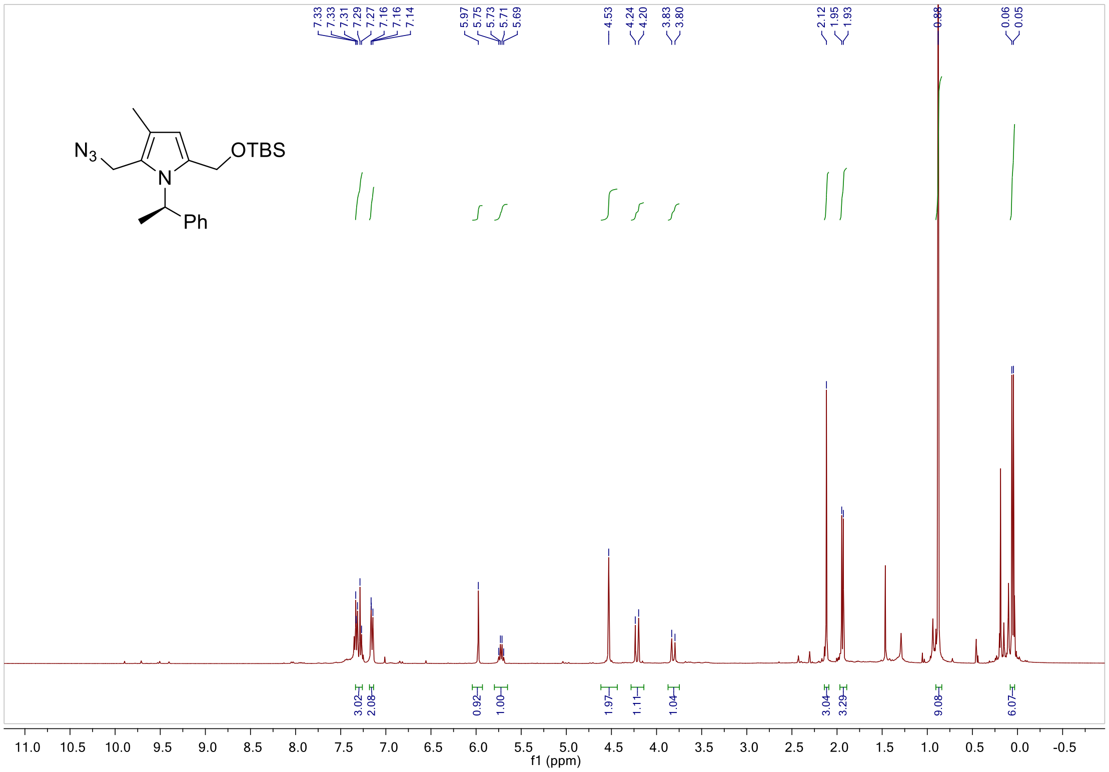

^{13}C NMR spectrum of (R)-2-(azidomethyl)-5-(((tert-butyldimethylsilyl)oxy)methyl)-3-methyl-1-(1-phenylethyl)-1H-pyrrole (2i):

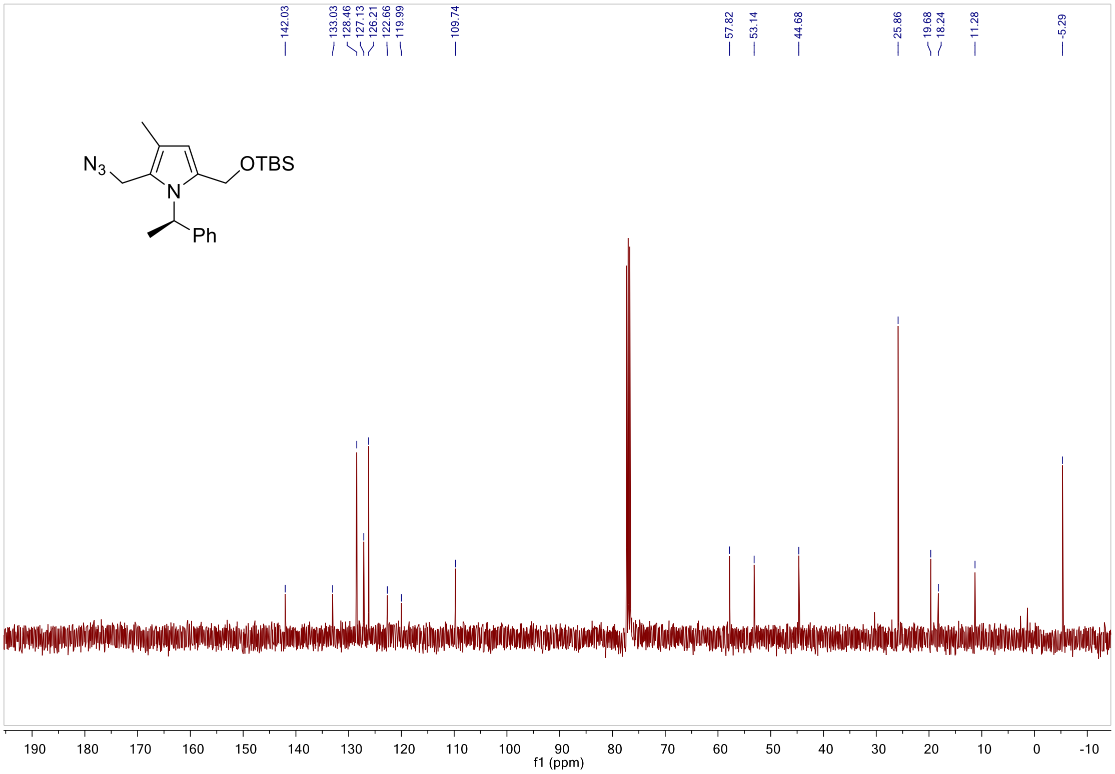

^{1}H NMR spectrum of (R)-2-(azidomethyl)-5-(((tert-butyldimethylsilyl)oxy)methyl)-3-(4-methoxyphenyl)-1-(1-phenylethyl)-1H-pyrrole (2j):

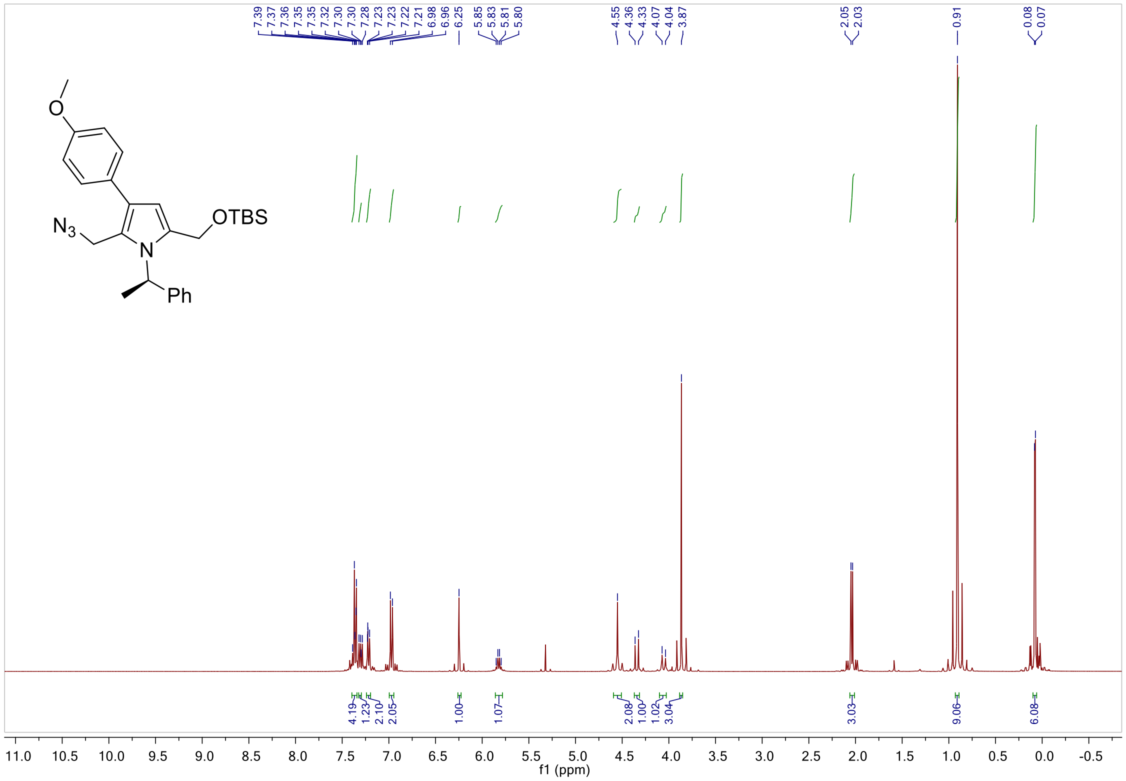

^{13}C NMR spectrum of (R)-2-(azidomethyl)-5-(((tert-butyldimethylsilyl)oxy)methyl)-3-(4-methoxyphenyl)-1-(1-phenylethyl)-1H-pyrrole (2j):

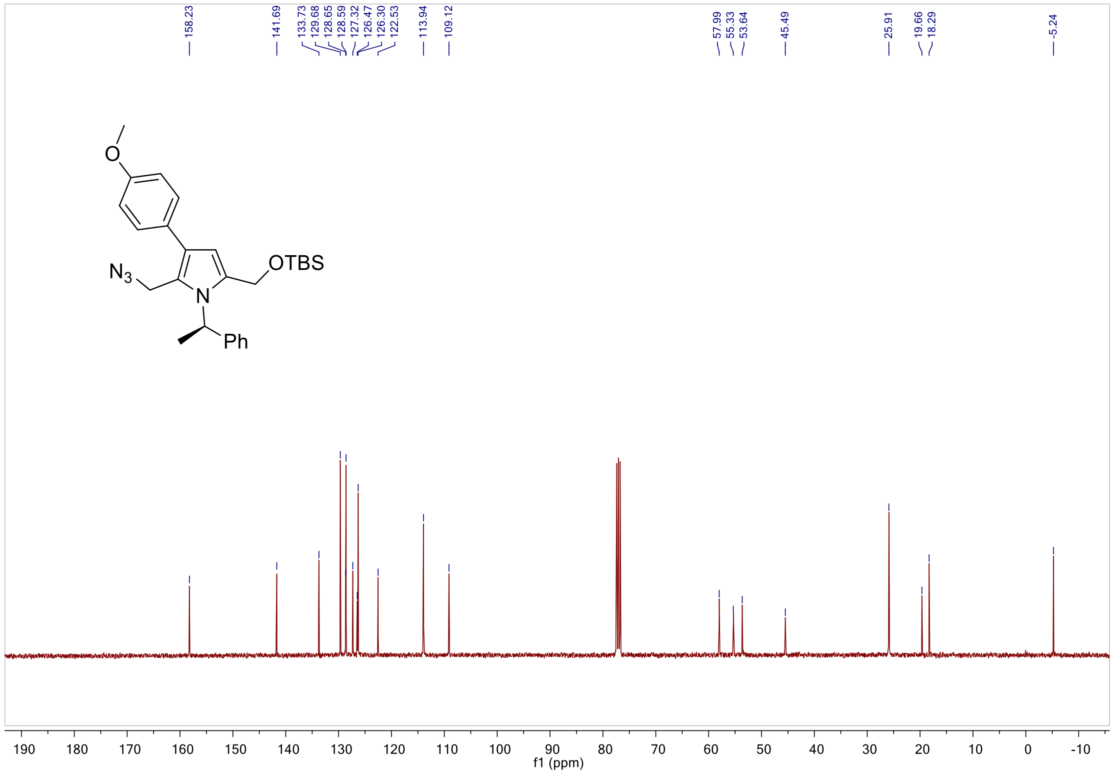

^{1}H NMR spectrum of (R)-5-(((5-(((tert-butyldimethylsilyl)oxy)methyl)-3-methyl-1-(1-phenylethyl)-1H-pyrrol-2-yl)methyl)thio)-1-phenyl-1H-tetrazole (2k):

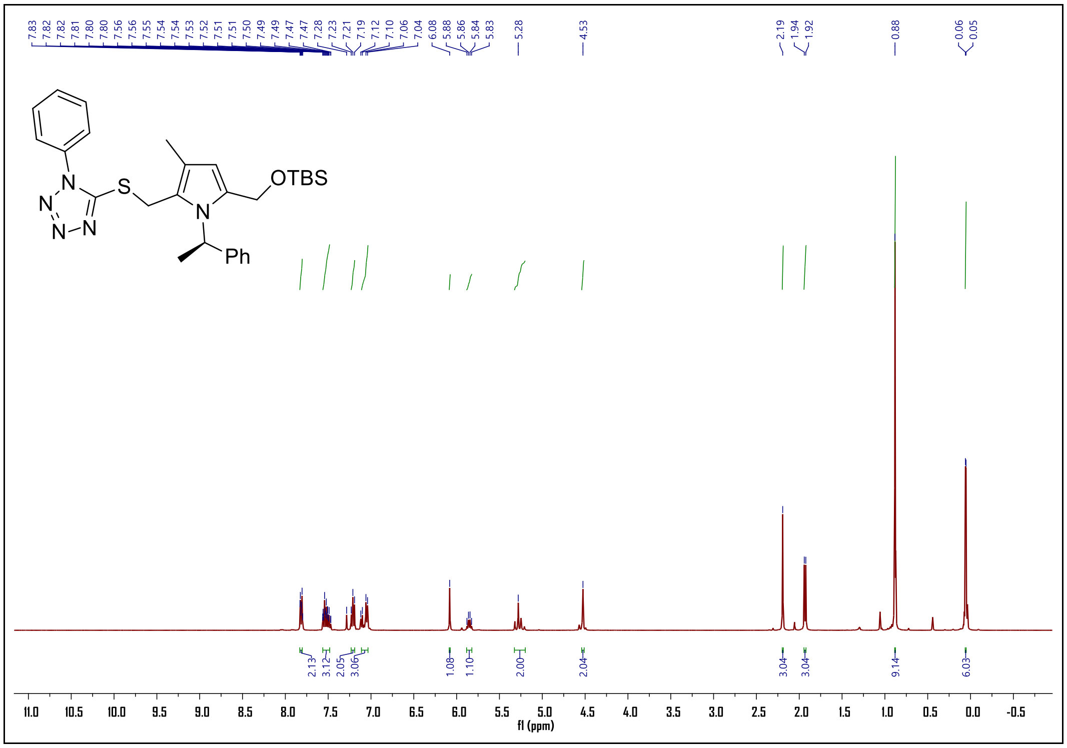

^{13}C NMR spectrum of (R)-5-(((5-(((tert-butyldimethylsilyl)oxy)methyl)-3-methyl-1-(1-phenylethyl)-1H-pyrrol-2-yl)methyl)thio)-1-phenyl-1H-tetrazole (2k):

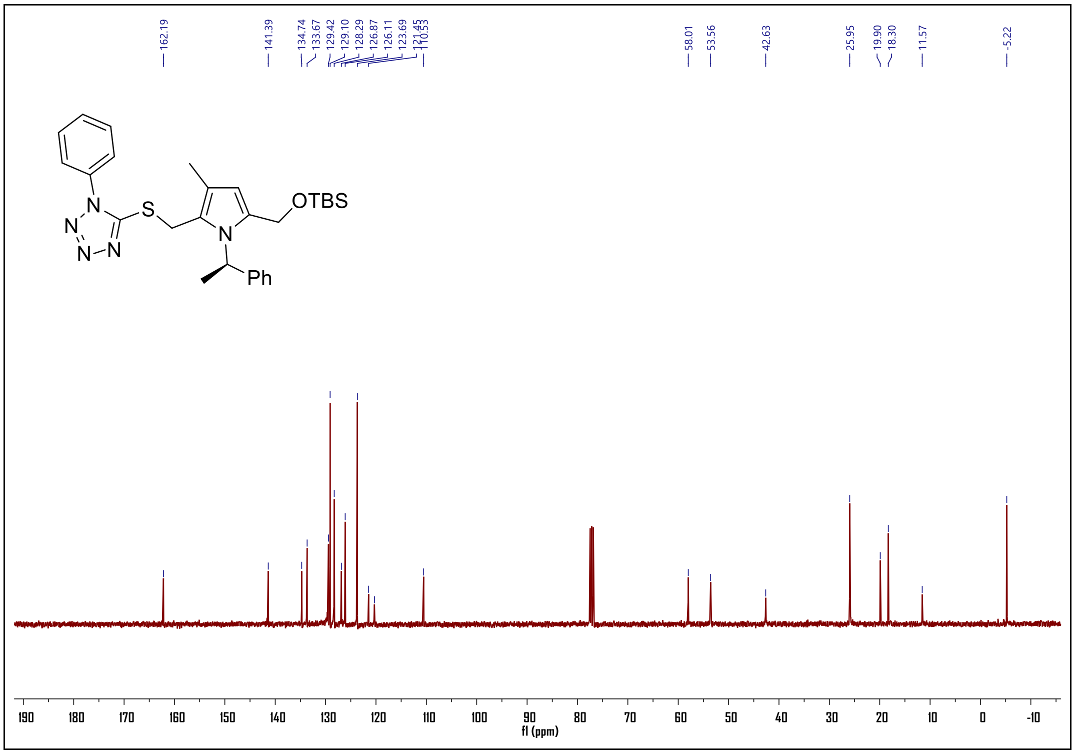

^{1}H NMR spectrum of 5-(((1-benzyl-5-nonyl-1H-pyrrol-2-yl)methyl)thio)-1-phenyl-1H-tetrazole (2l):

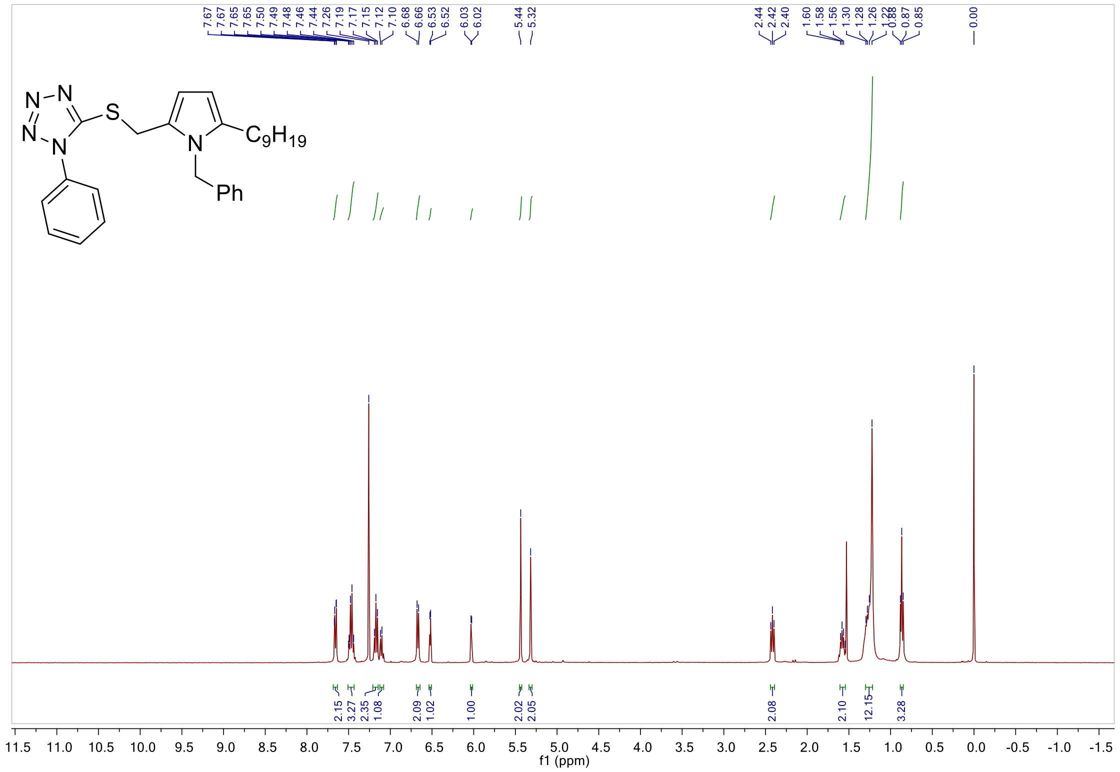

^{13}C NMR spectrum of 5-(((1-benzyl-5-nonyl-1H-pyrrol-2-yl)methyl)thio)-1-phenyl-1H-tetrazole (2l):

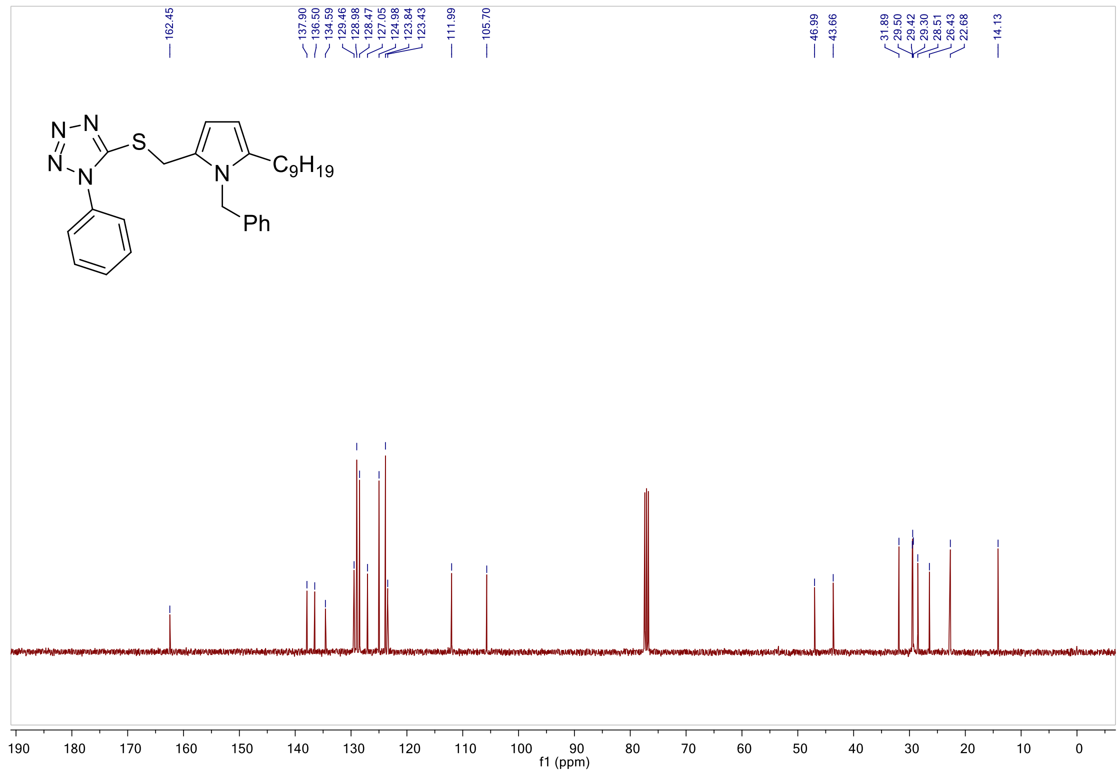

^{1}H NMR spectrum of 1-benzyl-2-(((4-methoxybenzyl)thio)methyl)-5-phenyl-1H-pyrrole (2m):

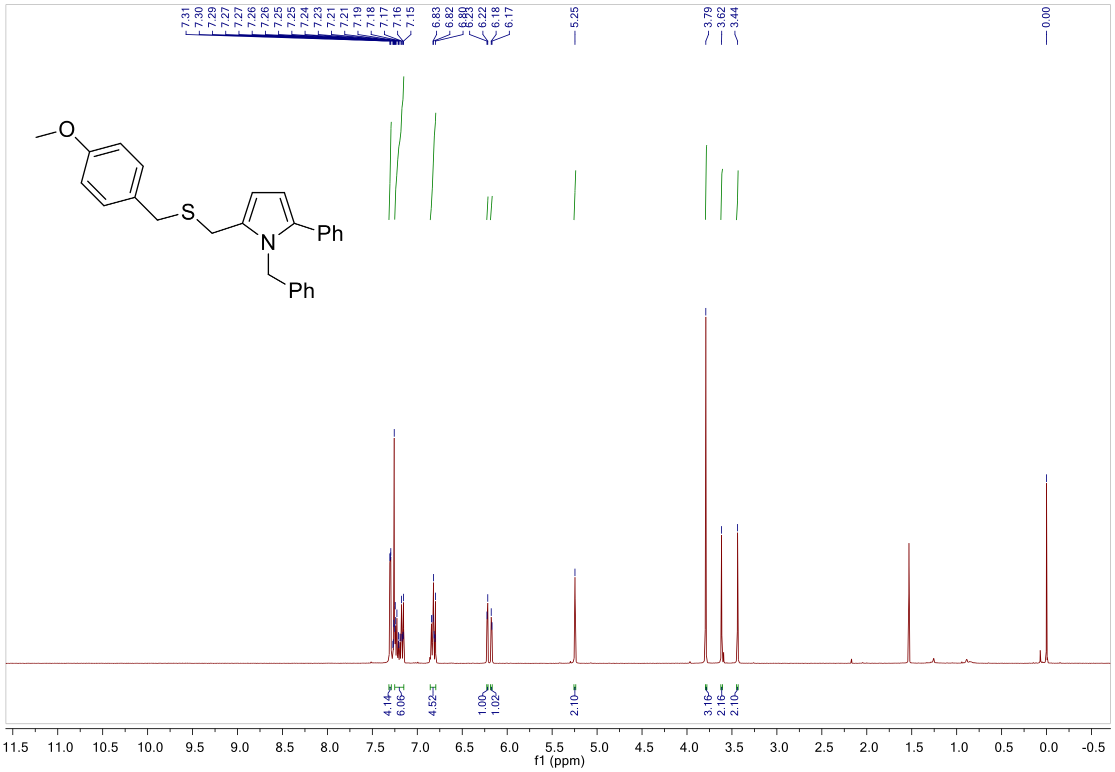

^{13}C NMR spectrum of 1-benzyl-2-(((4-methoxybenzyl)thio)methyl)-5-phenyl-1H-pyrrole (2m):

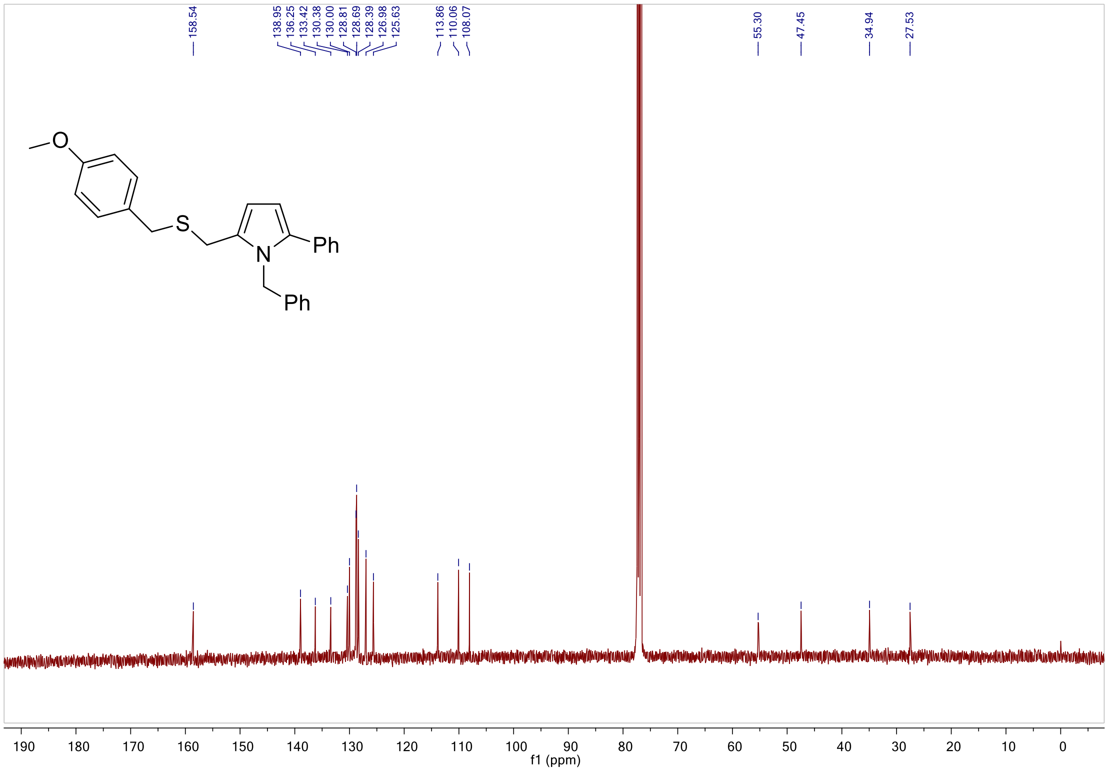

1H NMR spectrum of (R)-5-(methoxymethyl)-4-methyl-1-(1-phenylethyl)-1H-pyrrole-2-carbaldehyde (4a):

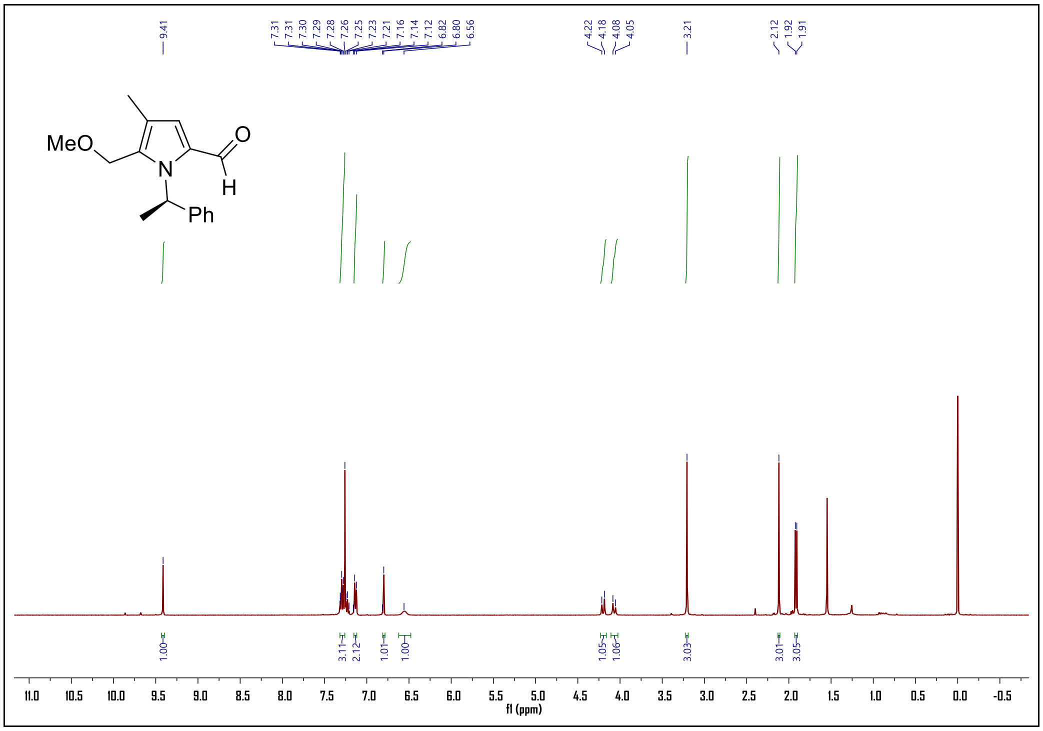

13C NMR spectrum of (R)-5-(methoxymethyl)-4-methyl-1-(1-phenylethyl)-1H-pyrrole-2-carbaldehyde (4a):

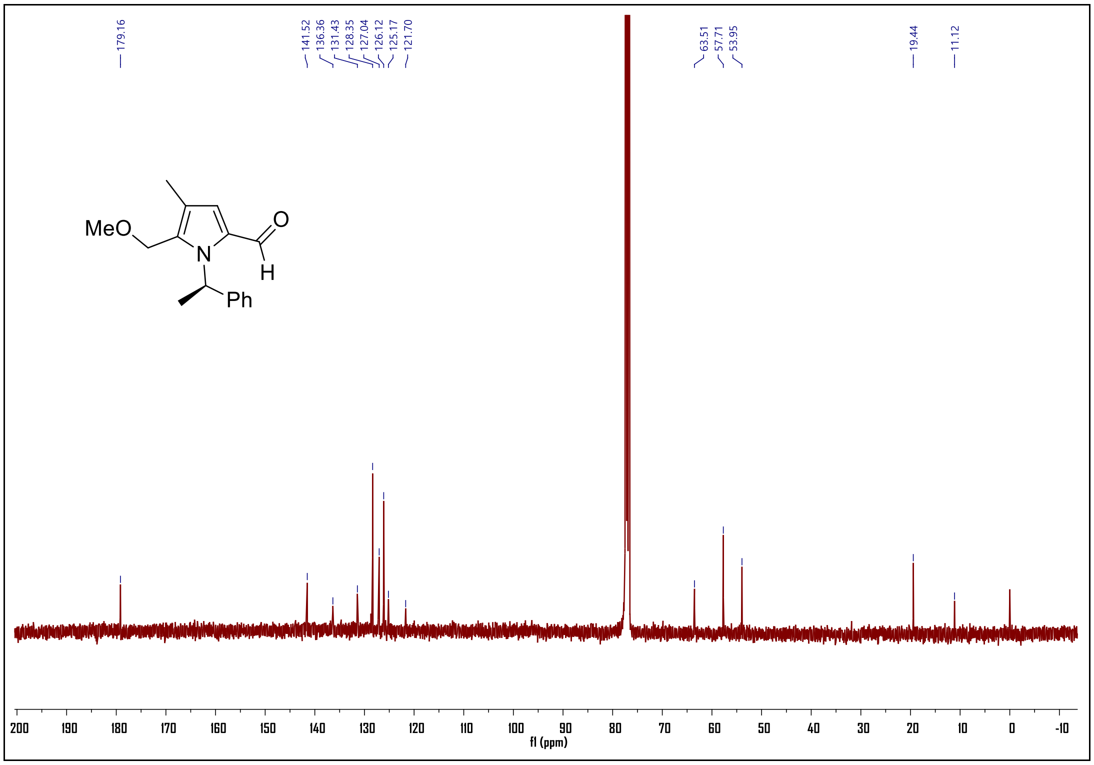

^{1}H NMR spectrum of (R)-5-(chloromethyl)-4-methyl-1-(1-phenylethyl)-1H-pyrrole-2-carbaldehyde (4b):

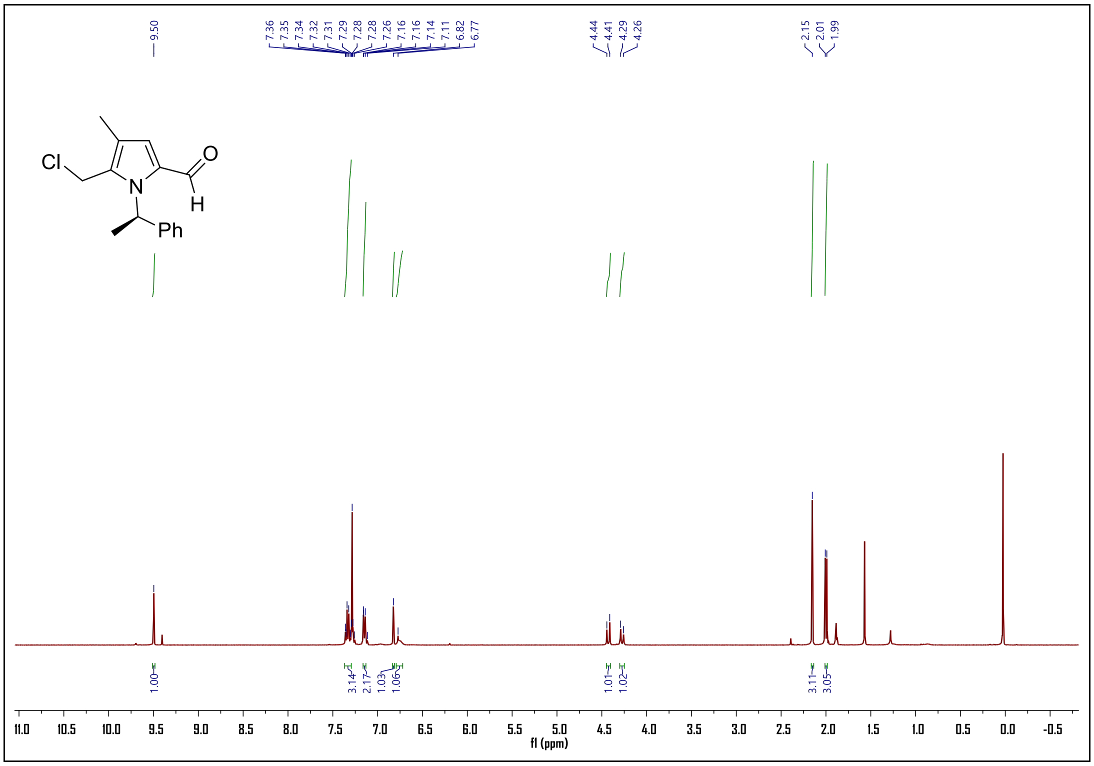

^{13}C NMR spectrum of (R)-5-(chloromethyl)-4-methyl-1-(1-phenylethyl)-1H-pyrrole-2-carbaldehyde (4b):

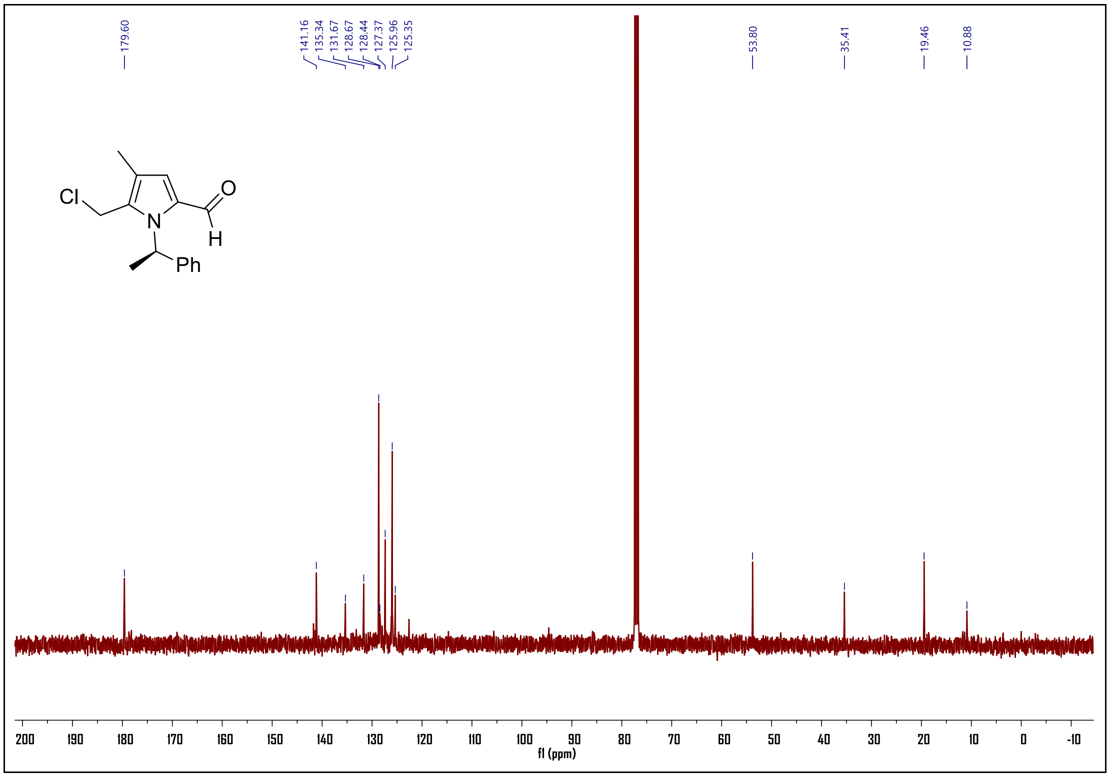

^{1}H NMR spectrum of (R)-(5-formyl-3-methyl-1-(1-phenylethyl)-1H-pyrrol-2-yl)methyl acetate (4c):

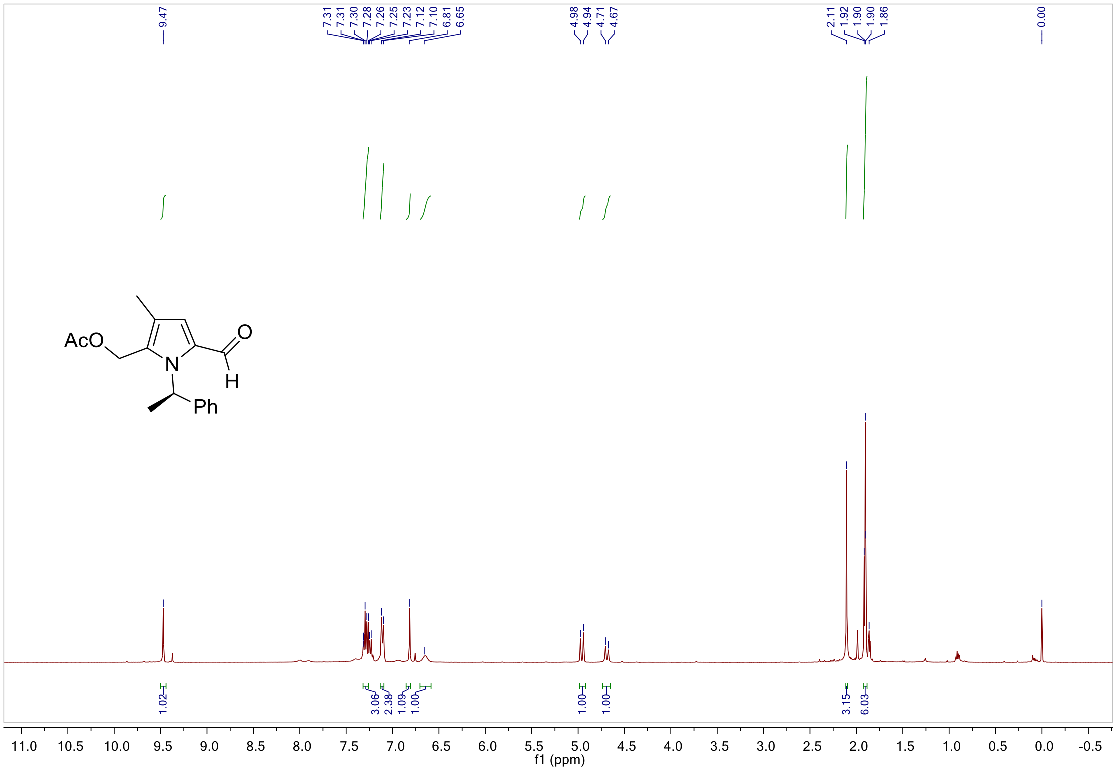

^{13}C NMR spectrum of (R)-(5-formyl-3-methyl-1-(1-phenylethyl)-1H-pyrrol-2-yl)methyl acetate (4c):

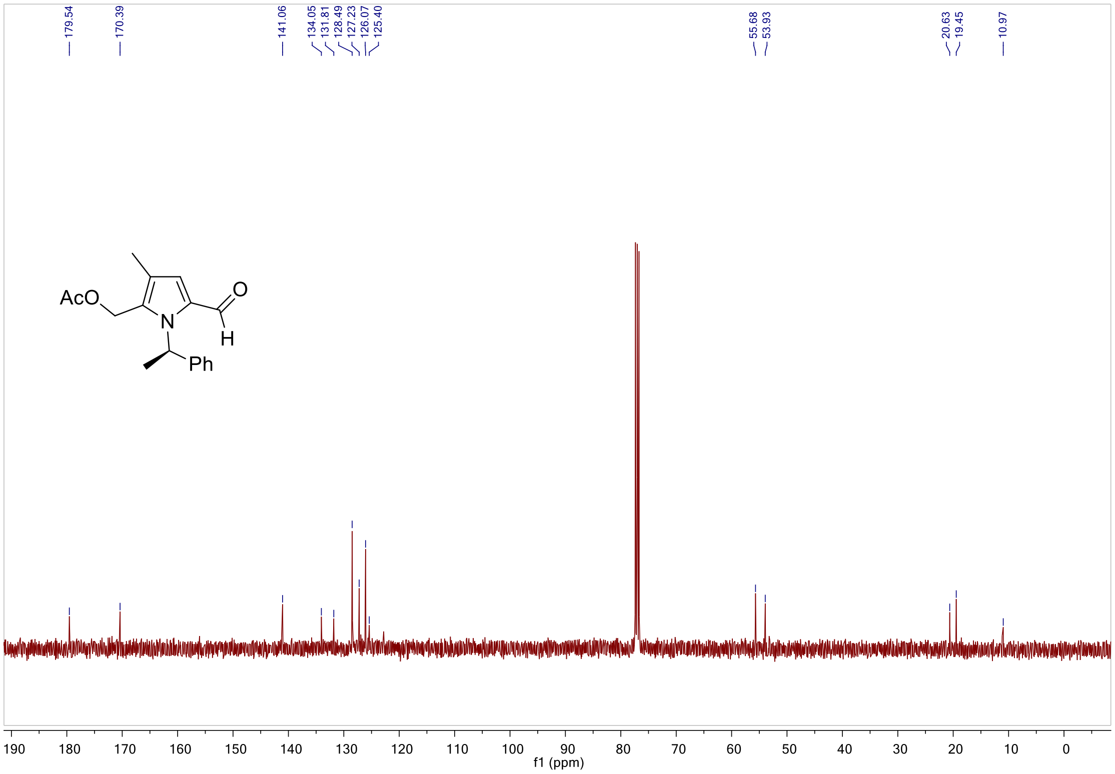

^{1}H NMR spectrum of (R)-2-(5-formyl-3-methyl-1-(1-phenylethyl)-1H-pyrrol-2-yl)acetonitrile (4d):

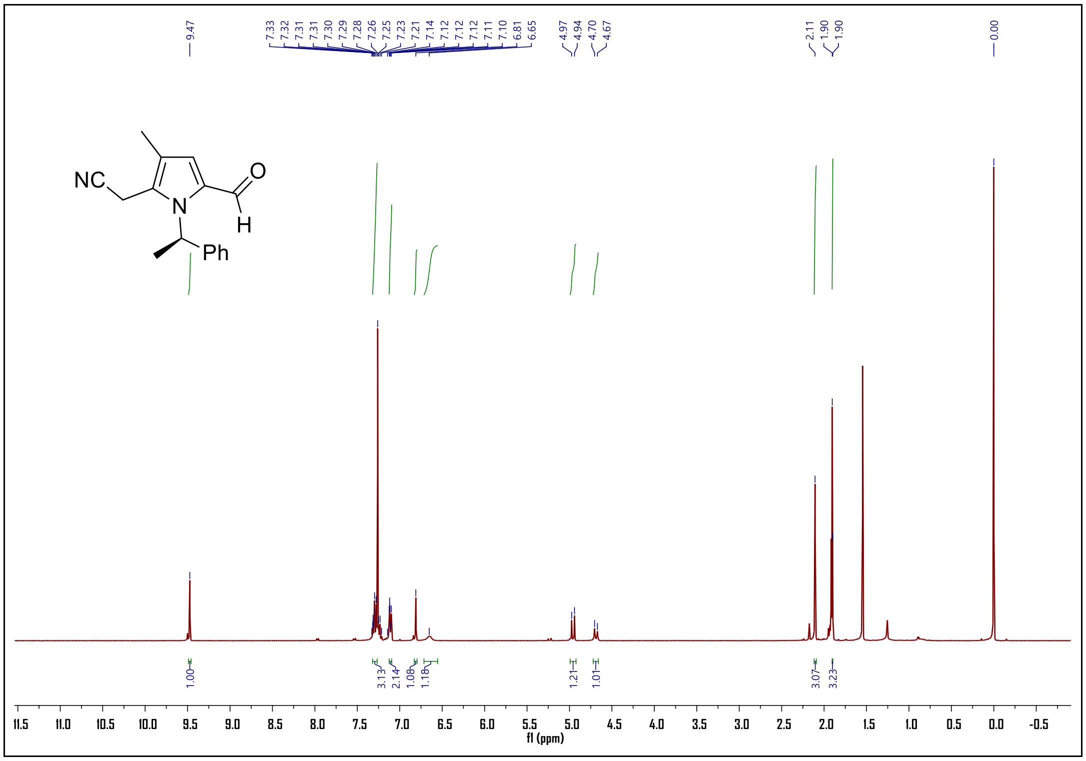

^{13}C NMR spectrum of (R)-2-(5-formyl-3-methyl-1-(1-phenylethyl)-1H-pyrrol-2-yl)acetonitrile (4d):

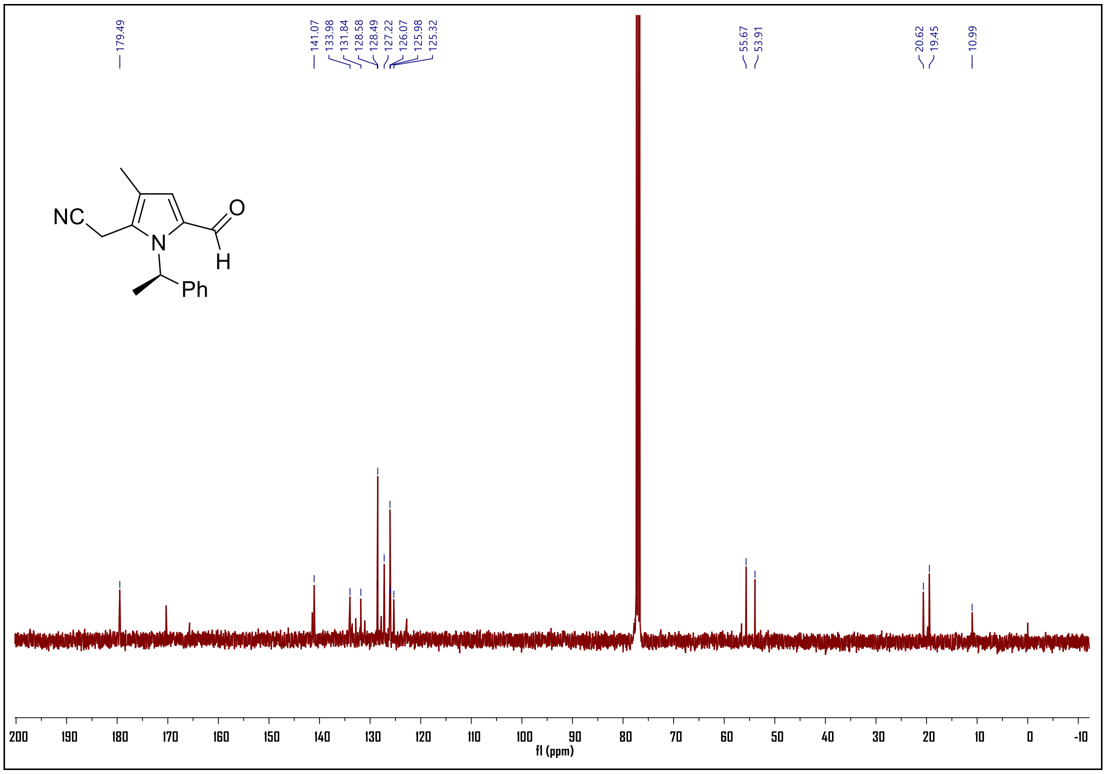

^{1}H NMR spectrum of Ethyl 1-phenethylaziridine-2-carboxylate (11):

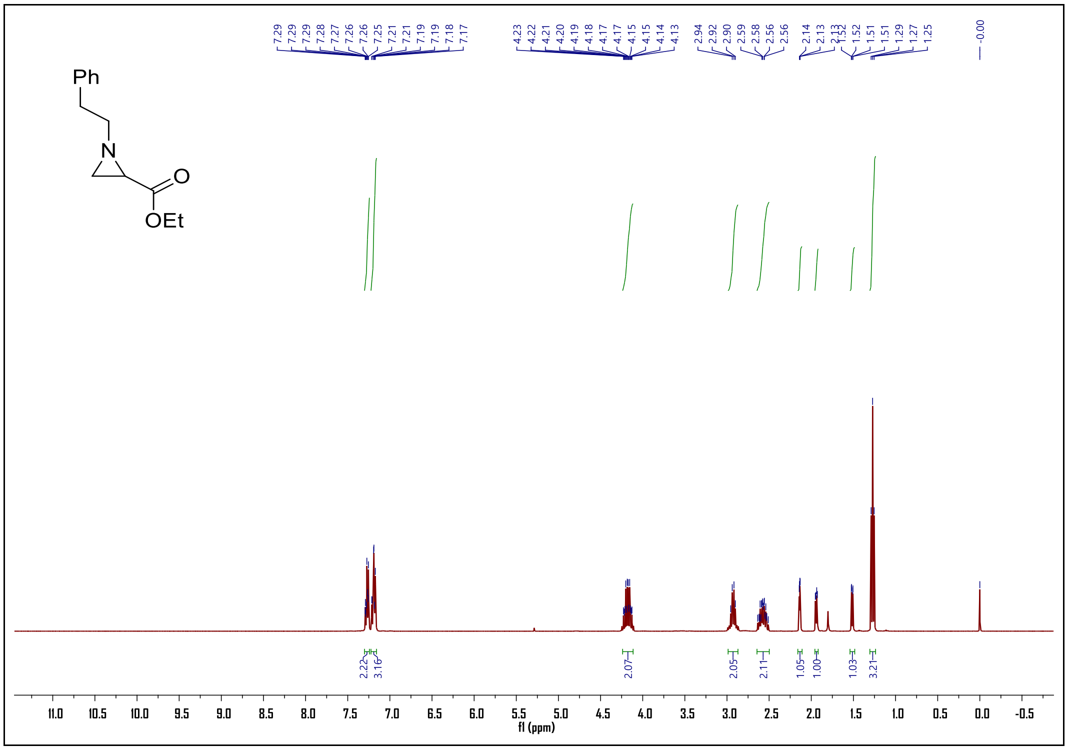

^{13}C NMR spectrum of Ethyl 1-phenethylaziridine-2-carboxylate (11):

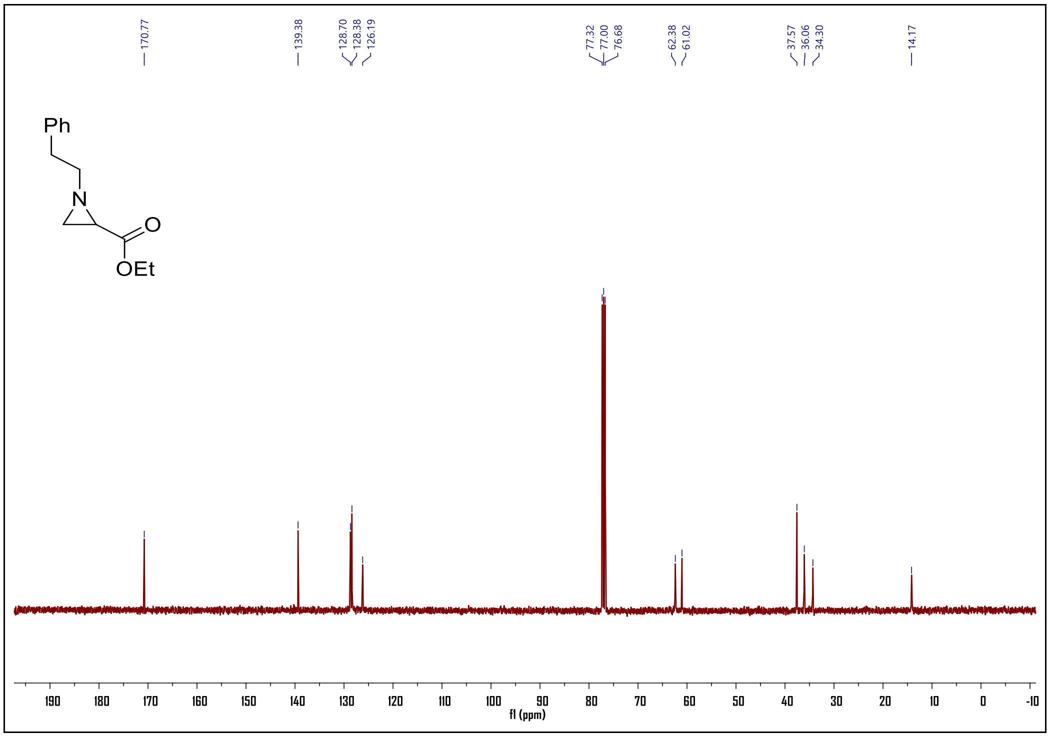

^{1}H NMR spectrum of N-mthoxy-N-methyl-1-phenethylaziridine-2-carboxamide (12):

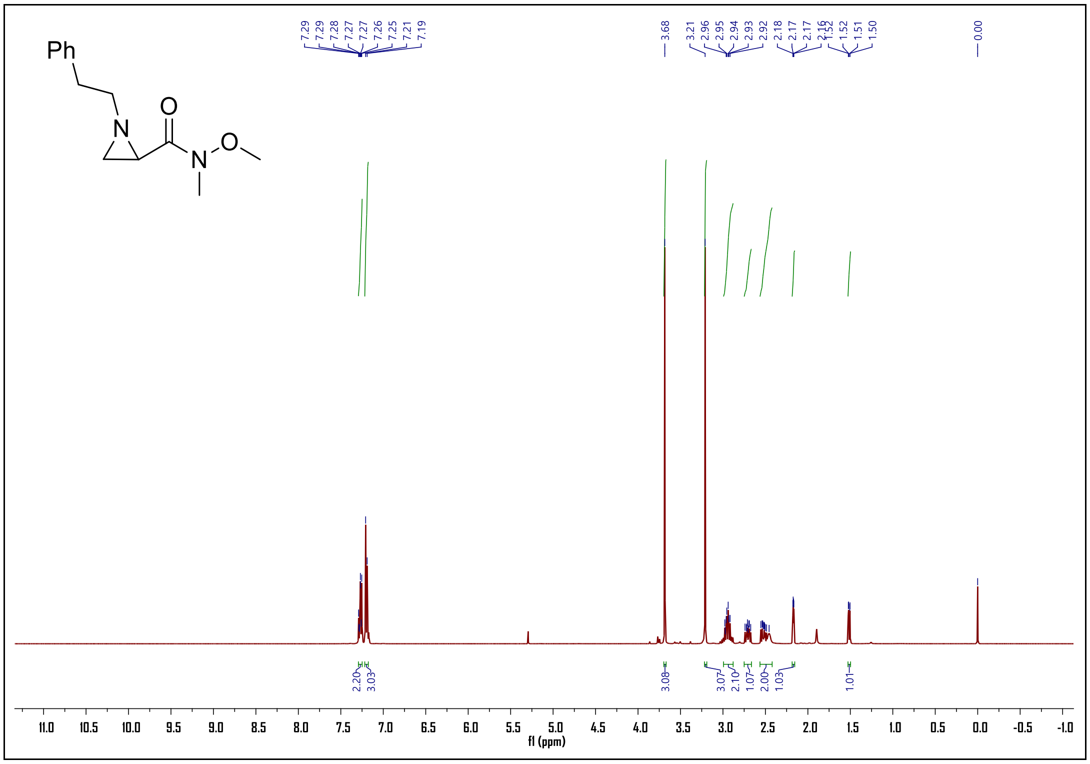

^{13}C NMR spectrum of N-mthoxy-N-methyl-1-phenethylaziridine-2-carboxamide (12):

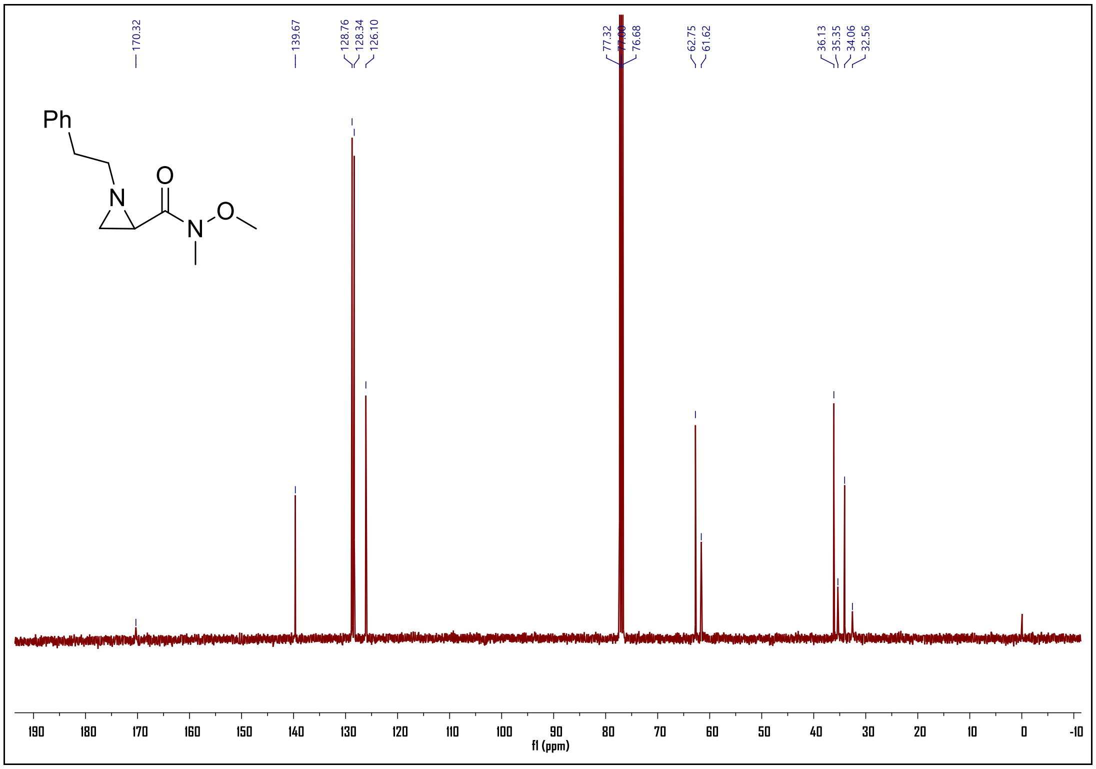

^{1}H NMR spectrum of 1-(1-phenethylaziridin-2-yl)but-3-en-1-ol (13):

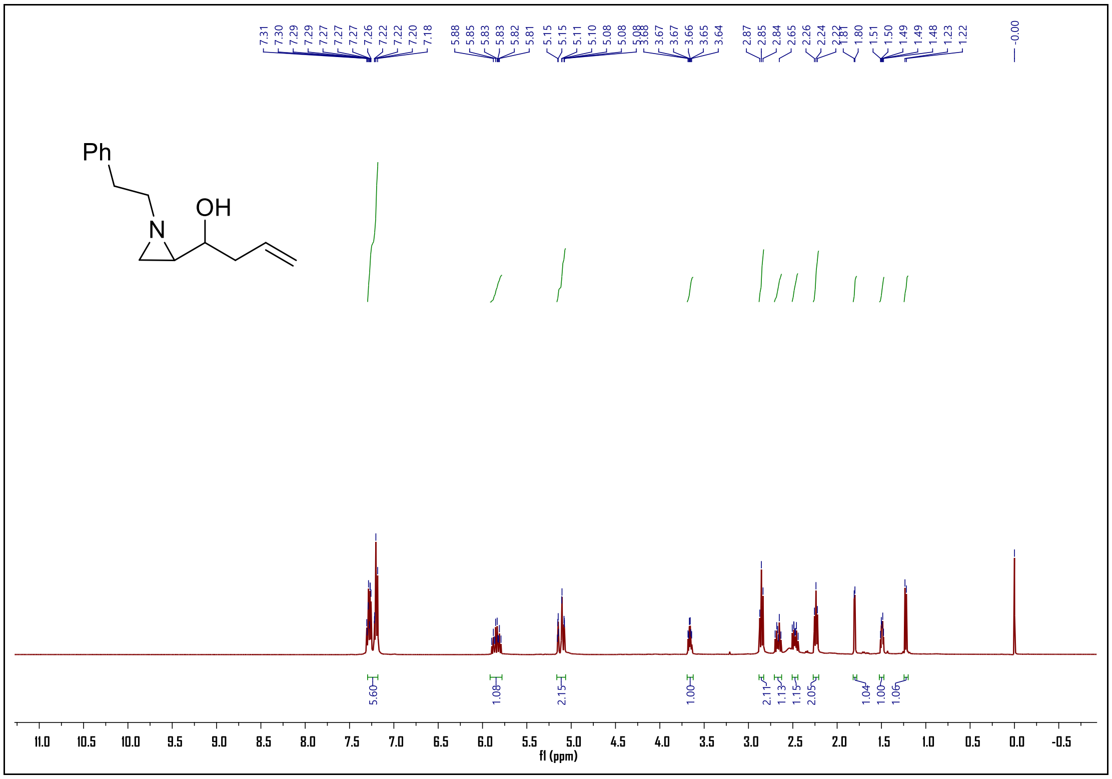

^{13}C NMR spectrum of 1-(1-phenethylaziridin-2-yl)but-3-en-1-ol (13):

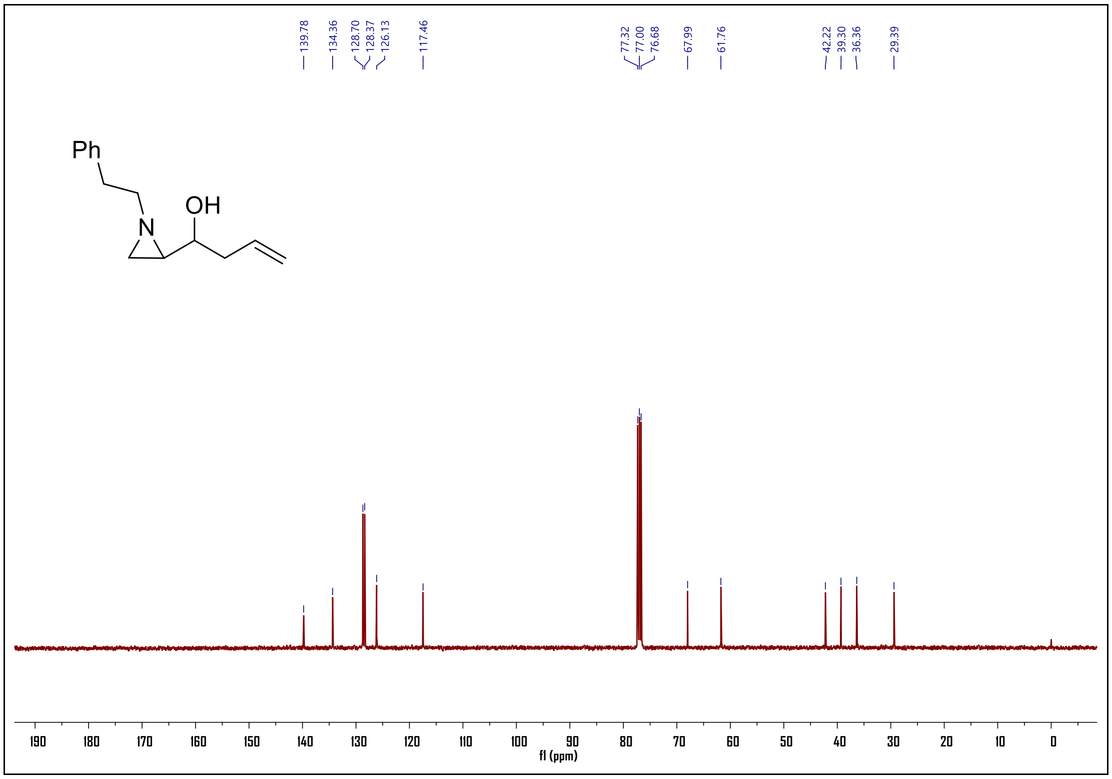

¹H NMR spectrum of 2-(1-((tert-butyldimethylsilyl)oxy)but-3-en-1-yl)-1-phenethylaziridine (14):

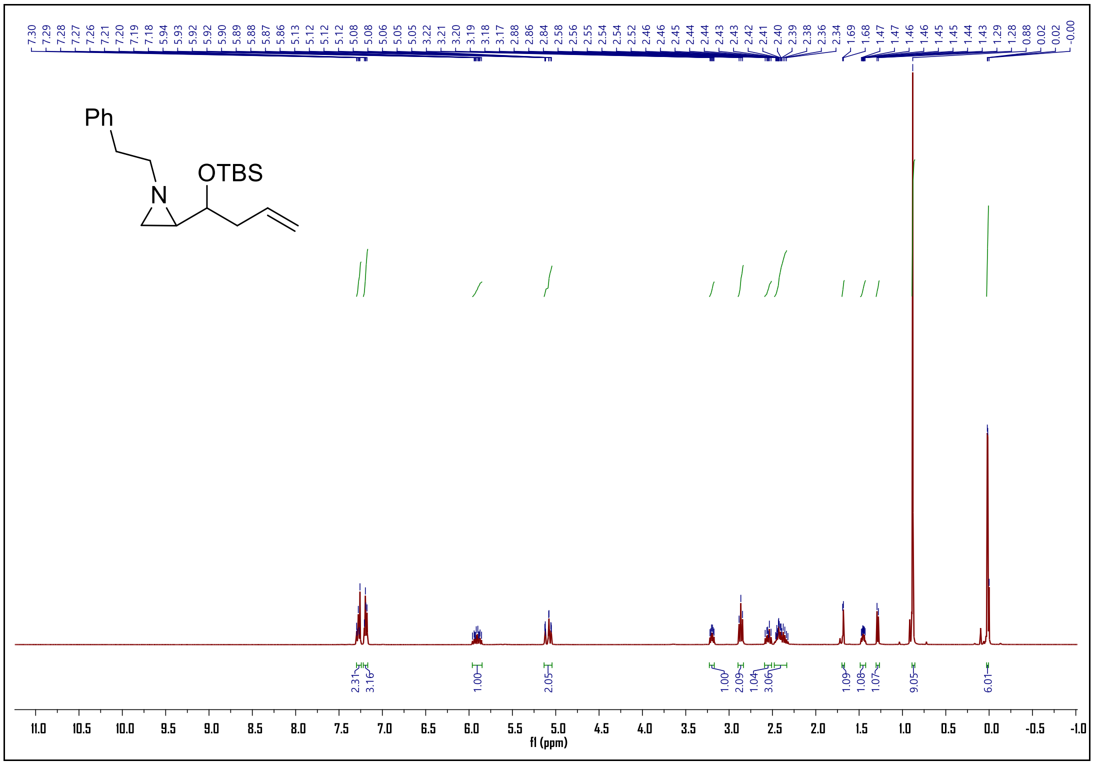

¹³C NMR spectrum of 2-(1-((tert-butyldimethylsilyl)oxy)but-3-en-1-yl)-1-phenethylaziridine (14):

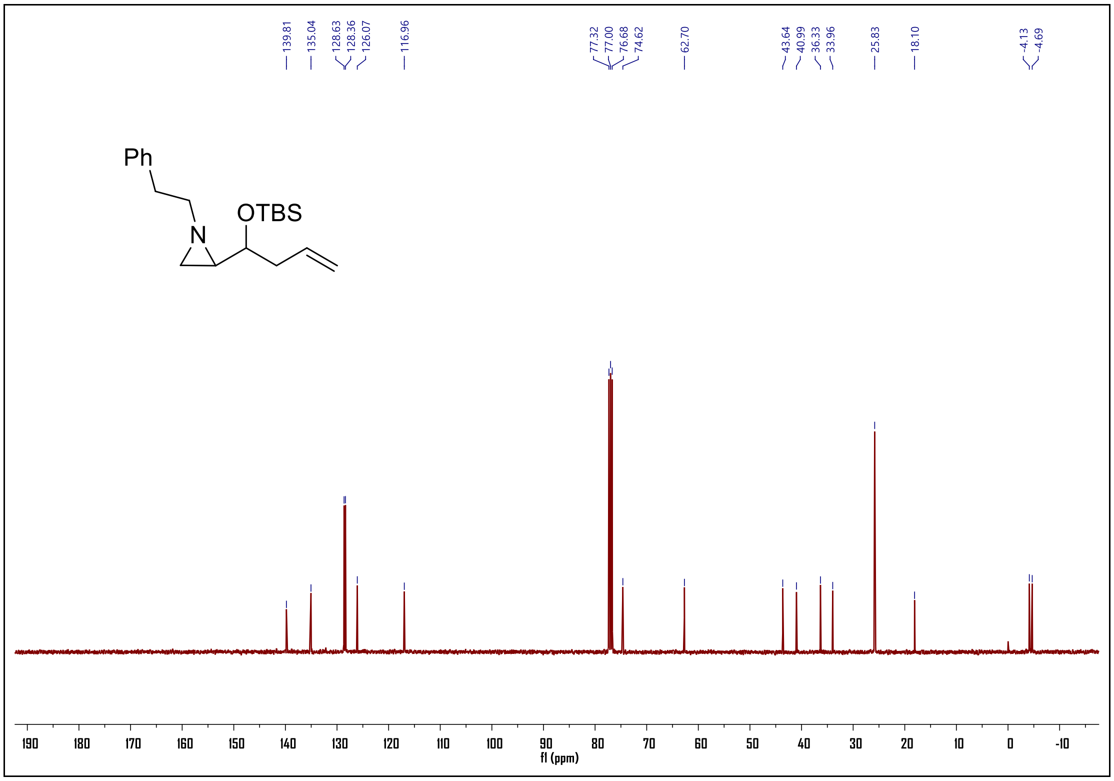

^{1}H NMR spectrum of Octamethyl-8-(1-phenethylaziridin-2-yl)-4,9-dioxa-3,10-disiladodecan-6-one (15):

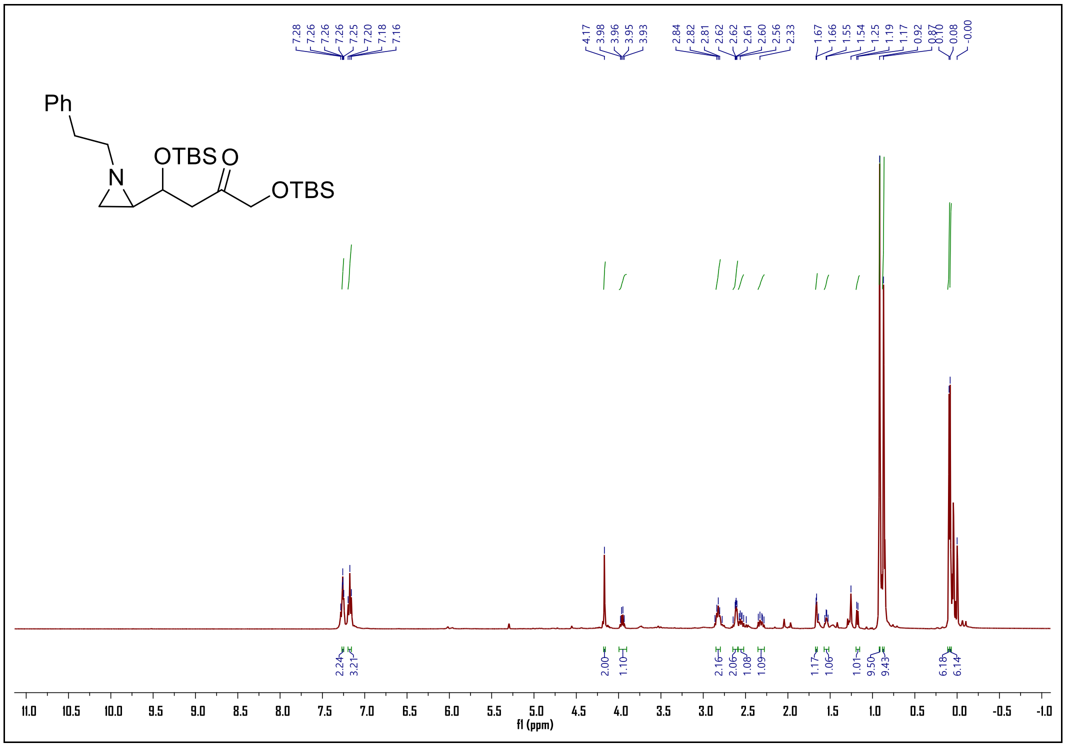

^{13}C NMR spectrum of Octamethyl-8-(1-phenethylaziridin-2-yl)-4,9-dioxa-3,10-disiladodecan-6-one (15):

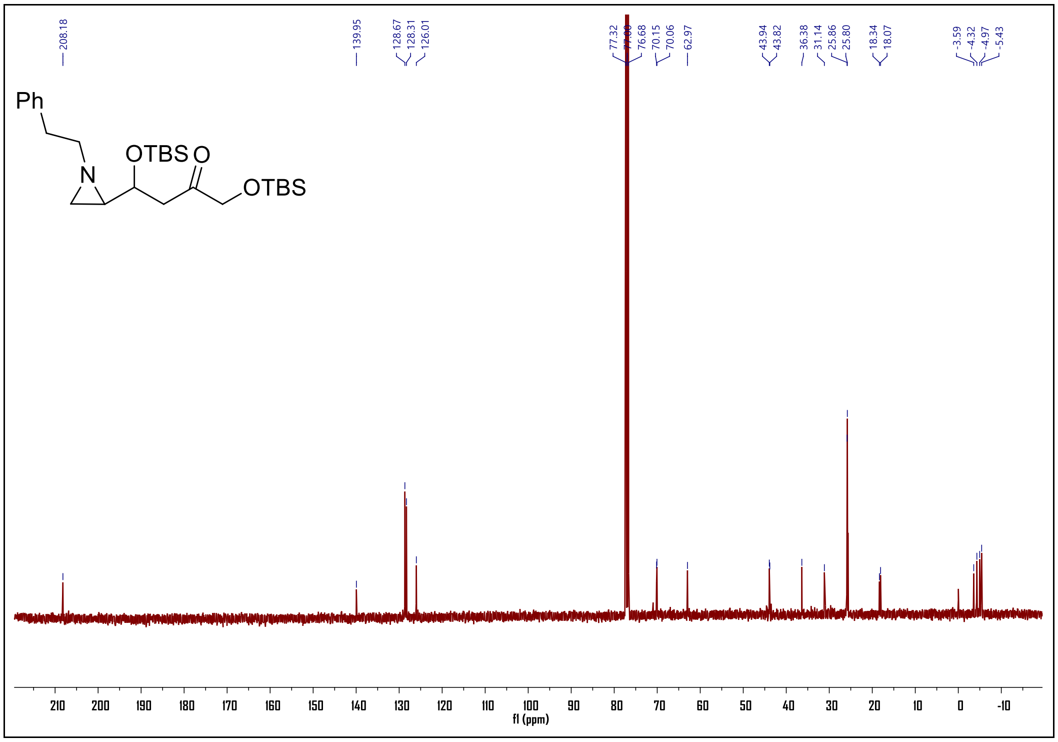

^{1}H NMR spectrum of (5-(((tert-butyldimethylsilyl)oxy)methyl)-1-phenethyl-1H-pyrrol-2-yl)methyl acetate (16):

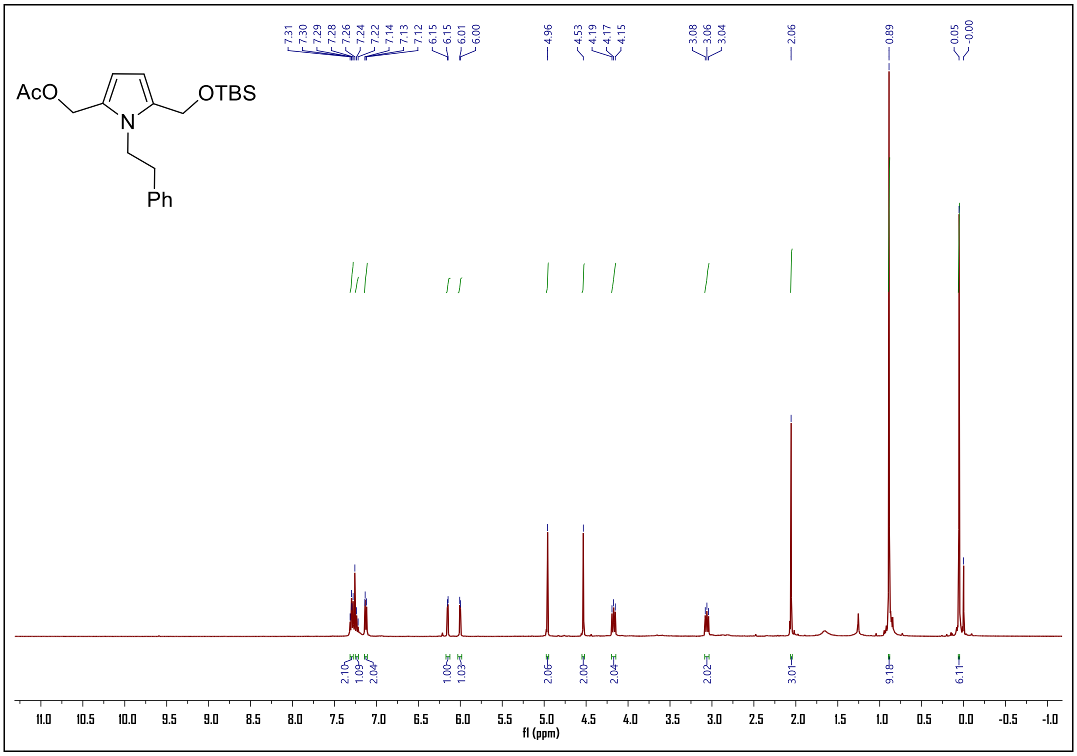

^{13}C NMR spectrum of (5-(((tert-butyldimethylsilyl)oxy)methyl)-1-phenethyl-1H-pyrrol-2-yl)methyl acetate (16):

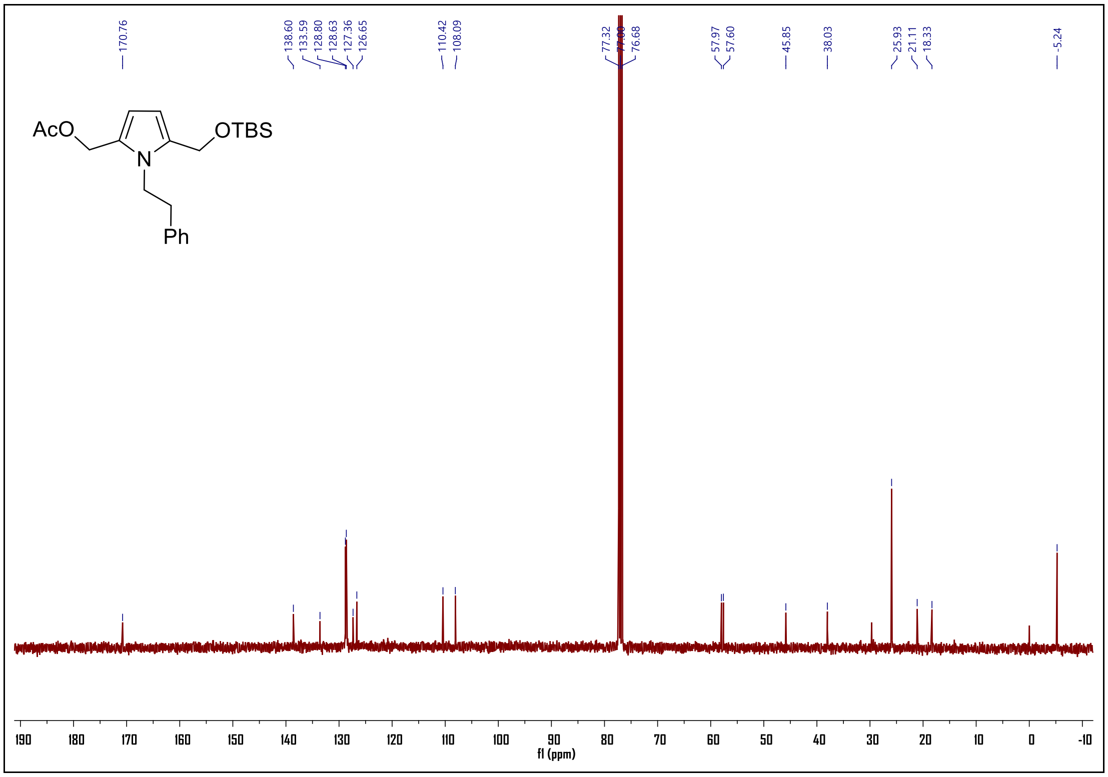

^{1}H NMR spectrum of (5-(((tert-butyldimethylsilyl)oxy)methyl)-1-phenethyl-1H-pyrrol-2-yl)methanol (17):

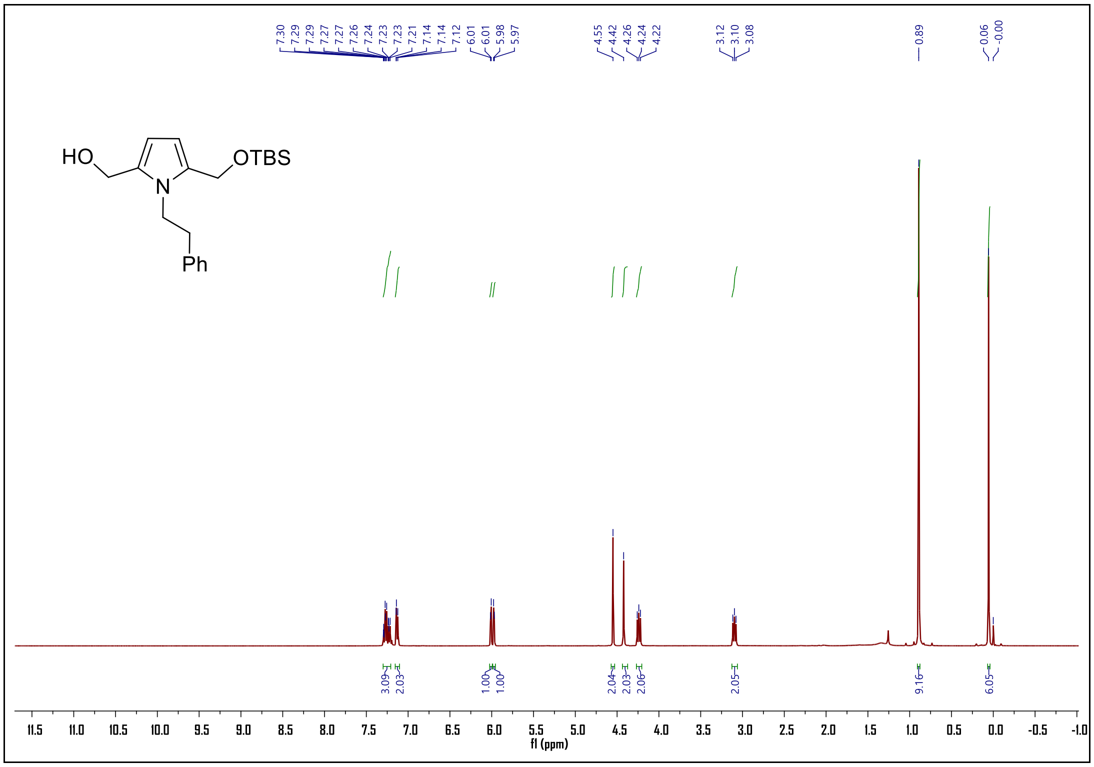

^{13}C NMR spectrum of (5-(((tert-butyldimethylsilyl)oxy)methyl)-1-phenethyl-1H-pyrrol-2-yl)methanol (17):

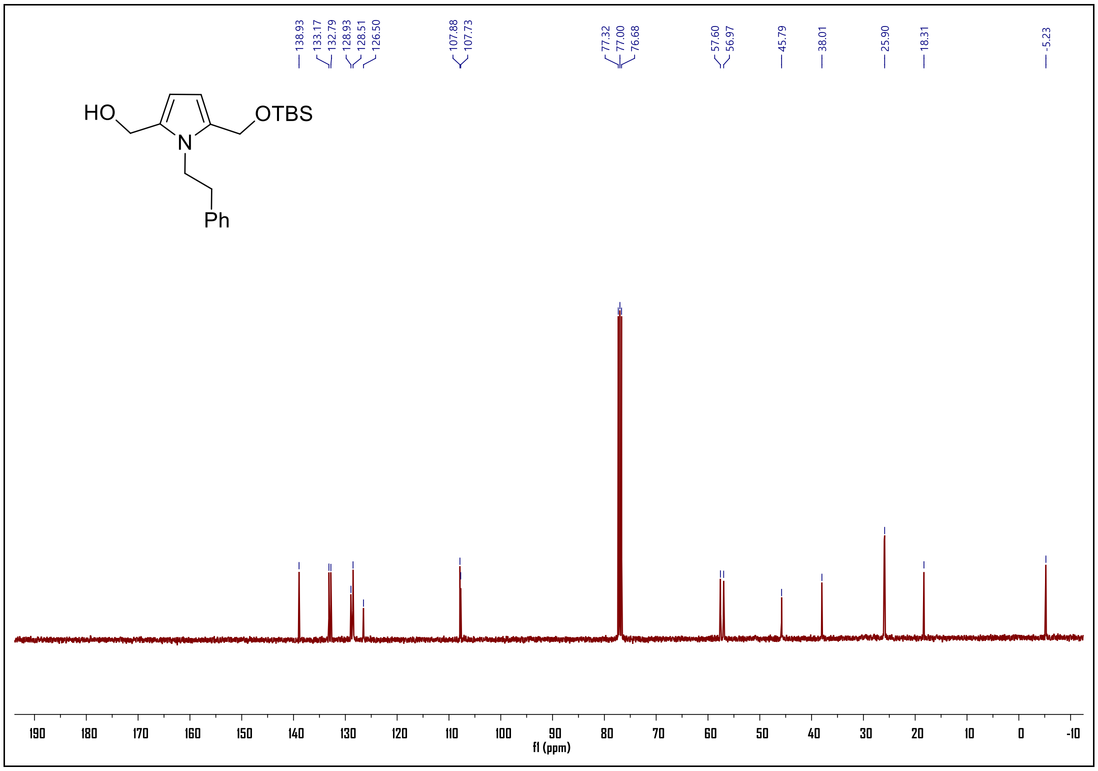

^{1}H NMR spectrum of 5-(((tert-butyldimethylsilyl)oxy)methyl)-1-phenethyl-1H-pyrrole-2-carbaldehyde (18):

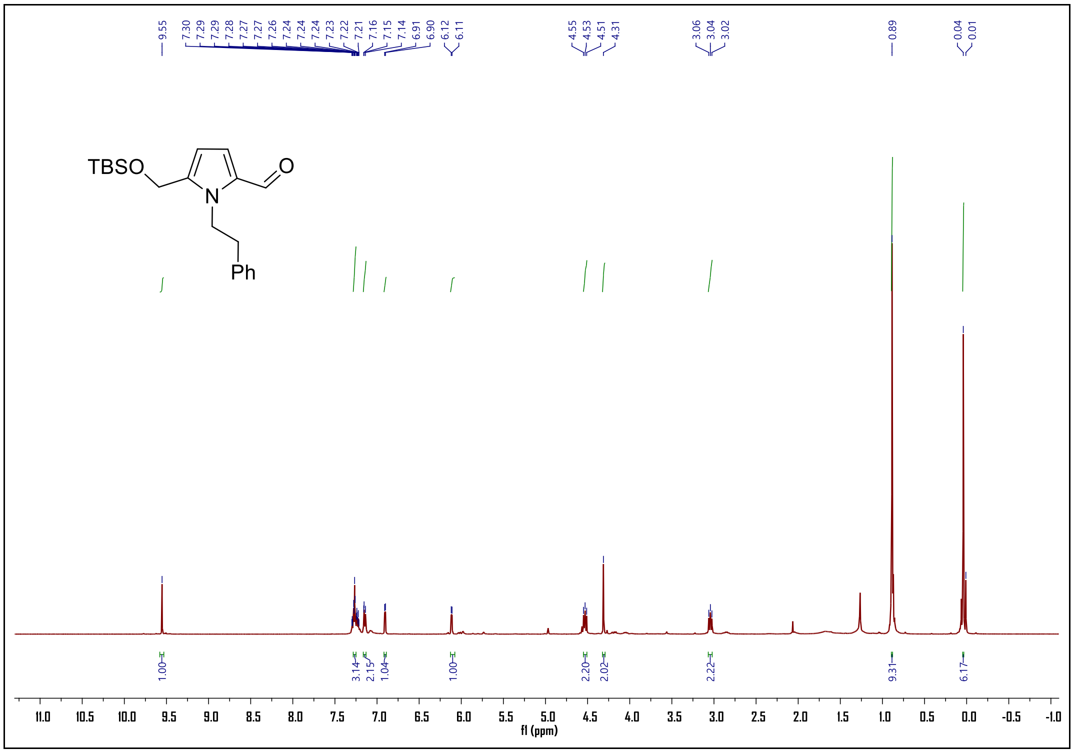

^{13}C NMR spectrum of 5-(((tert-butyldimethylsilyl)oxy)methyl)-1-phenethyl-1H-pyrrole-2-carbaldehyde (18):

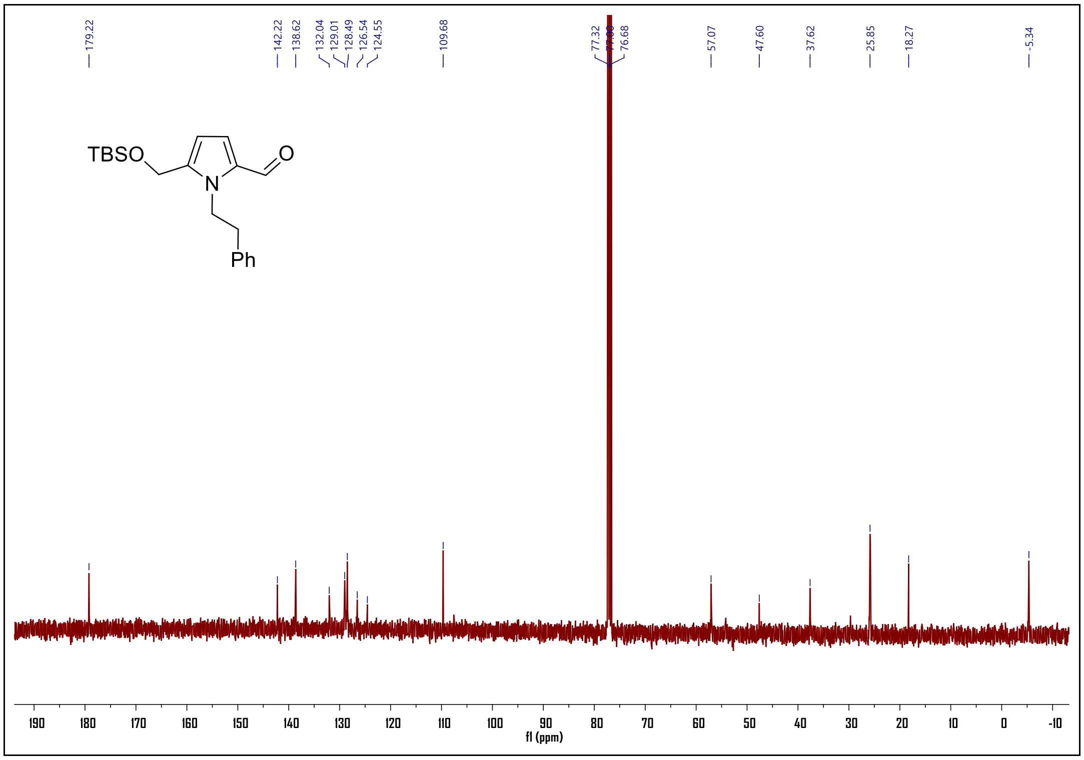

^{1}H NMR spectrum of 5-(hydroxymethyl)-1-phenethyl-1H-pyrrole-2-carbaldehyde (19):

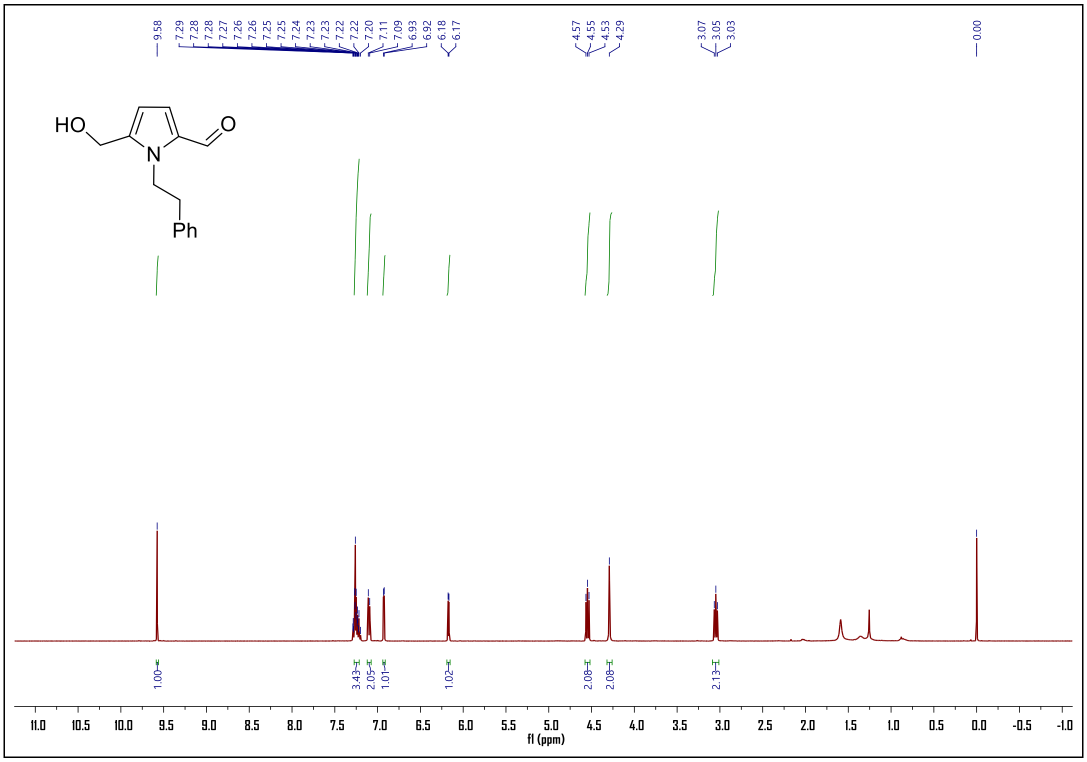

^{13}C NMR spectrum of 5-(hydroxymethyl)-1-phenethyl-1H-pyrrole-2-carbaldehyde (19):

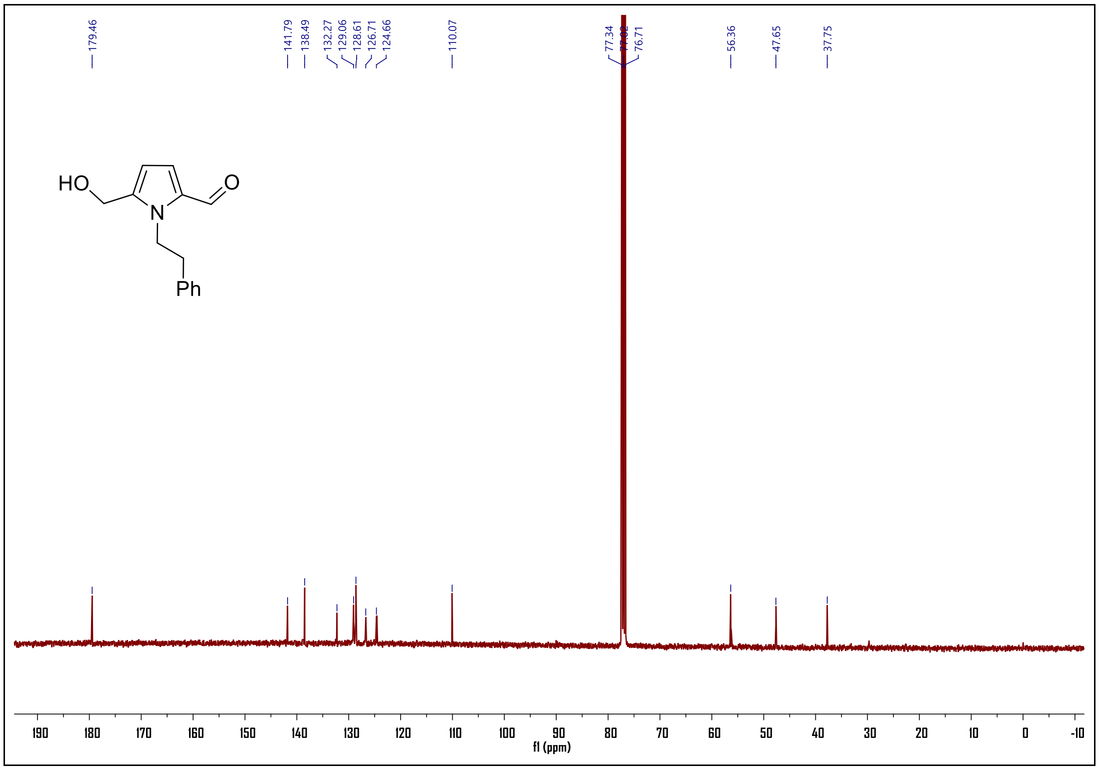

Supplement: Supplementary file 1 [file molecules-27-06869-s001.zip › molecules-1967828-supplementary.pdf]
